# From algae to angiosperms–inferring the phylogeny of green plants (*Viridiplantae*) from 360 plastid genomes

**DOI:** 10.1186/1471-2148-14-23

**Published:** 2014-02-17

**Authors:** Brad R Ruhfel, Matthew A Gitzendanner, Pamela S Soltis, Douglas E Soltis, J Gordon Burleigh

**Affiliations:** 1 Department of Biological Sciences, Eastern Kentucky University, Richmond, KY 40475, USA; 2 Department of Biology, University of Florida, Gainesville, FL 32611-8525, USA; 3 Florida Museum of Natural History, University of Florida, Gainesville, FL 32611-7800, USA; 4 Genetics Institute, University of Florida, Gainesville, FL 32610, USA

**Keywords:** Composition bias, Phylogenomics, Plastid genome sequences, Plastomes, RY-coding, *Viridiplantae*

## Abstract

**Background:**

Next-generation sequencing has provided a wealth of plastid genome sequence data from an increasingly diverse set of green plants (*Viridiplantae*). Although these data have helped resolve the phylogeny of numerous clades (e.g., green algae, angiosperms, and gymnosperms), their utility for inferring relationships across all green plants is uncertain. *Viridiplantae* originated 700-1500 million years ago and may comprise as many as 500,000 species. This clade represents a major source of photosynthetic carbon and contains an immense diversity of life forms, including some of the smallest and largest eukaryotes. Here we explore the limits and challenges of inferring a comprehensive green plant phylogeny from available complete or nearly complete plastid genome sequence data.

**Results:**

We assembled protein-coding sequence data for 78 genes from 360 diverse green plant taxa with complete or nearly complete plastid genome sequences available from GenBank. Phylogenetic analyses of the plastid data recovered well-supported backbone relationships and strong support for relationships that were not observed in previous analyses of major subclades within *Viridiplantae*. However, there also is evidence of systematic error in some analyses. In several instances we obtained strongly supported but conflicting topologies from analyses of nucleotides versus amino acid characters, and the considerable variation in GC content among lineages and within single genomes affected the phylogenetic placement of several taxa.

**Conclusions:**

Analyses of the plastid sequence data recovered a strongly supported framework of relationships for green plants. This framework includes: i) the placement of *Zygnematophyceace* as sister to land plants (*Embryophyta*), ii) a clade of extant gymnosperms (*Acrogymnospermae*) with cycads + *Ginkgo* sister to remaining extant gymnosperms and with gnetophytes (*Gnetophyta*) sister to non-*Pinaceae* conifers (Gnecup trees), and iii) within the monilophyte clade (*Monilophyta*), *Equisetales* + *Psilotales* are sister to *Marattiales* + leptosporangiate ferns. Our analyses also highlight the challenges of using plastid genome sequences in deep-level phylogenomic analyses, and we provide suggestions for future analyses that will likely incorporate plastid genome sequence data for thousands of species. We particularly emphasize the importance of exploring the effects of different partitioning and character coding strategies.

## Background

*Viridiplantae*, or green plants, are a clade of perhaps 500,000 species [1-6] that exhibit an astounding diversity of life forms, including some of the smallest and largest eukaryotes [3,7]. Fossil evidence suggests the clade is at least 750 million years old [8-10], while divergence time estimates from molecular data suggest it may be more than one billion years old [11-14]. Reconstructing the phylogenetic relationships across green plants is challenging because of the age of the clade, the extinction of major lineages [15-17], and extreme molecular rate and compositional heterogeneity [18-22]. Most phylogenetic analyses of *Viridiplantae* have recovered two well-supported subclades, *Chlorophyta* and *Streptophyta*[23,24]. *Chlorophyta* contain most of the traditionally recognized “green algae,” and *Streptophyta* contain the land plants (*Embryophyta*), as well as several other lineages also considered “green algae”. Land plants include the seed plants (gymnosperms and angiosperms; *Spermatophyta*), which consist of ~270,000 to ~450,000 species [1,3].

While many of the major green plant clades are well defined, questions remain regarding the relationships among them. For example, the closest relatives of land plants have varied among analyses [23,25-29], as have the relationships among the three bryophyte lineages (mosses, liverworts, and hornworts) [29-35]. The relationships among extant gymnosperms also remain contentious, particularly with respect to the placement of *Gnetophyta*[20,36-43].

Most broad analyses of green plant relationships based on nuclear gene sequence data have relied largely on 18S/26S rDNA sequences [30,37,44,45], although recent analyses have employed numerous nuclear genes [40,46]. Some studies have used mitochondrial gene sequence data, often in combination with other data [29,47,48]. However, investigations of green plant phylogeny typically have either largely or exclusively employed chloroplast genes (e.g., [29,49-52]). Sequence data from the plastid genome have transformed plant systematics and contributed greatly to the current view of plant relationships. With the plastid genome present in high copy numbers in each cell in most plants, and with relatively little variation in gene content and order [53], as well as few reported instances of gene duplication or horizontal gene transfer [54,55], the plastid genome provides a wealth of phylogenetically informative data that are relatively easy to obtain and use [56,57]. Although early phylogenetic studies using one or a few chloroplast loci provided fundamental insights into relationships within and among green plant clades, these analyses failed to resolve some backbone relationships [56-59]. These remaining enigmatic portions of the green plant tree of life ultimately motivated the use of entire, or nearly entire, plastid genome sequences for phylogenetic inference.

Complete sequencing of the relatively small (~150 kb) plastid genome has been technically feasible since the mid-1980s [60,61], although few plastid genomes were sequenced prior to 2000 (see [62,63]). Next-generation sequencing (NGS) technologies, such as 454 [62] and Illumina [64-67], greatly reduced the cost and difficulty of sequencing plastid genomes, and consequently, the number of plastid genomes available on GenBank increased nearly six-fold from 2006 to 2012 [68]. Phylogenetic analyses based on complete plastid genome sequences have provided valuable insights into relationships among and within subclades across the green plant tree of life (recently reviewed in [26,35,68,69]). Still, studies employing complete plastid genomes generally have either focused on subclades of green plants or have had relatively low taxon sampling. Thus, they have not addressed the major relationships across all green plants simultaneously.

We assembled available plastid genome sequences to build a phylogenetic framework for *Viridiplantae* that reflects the wealth of new plastid genome sequence data. Furthermore, we highlight analytical challenges for resolving the green plant tree of life with this type of data. We performed phylogenetic analyses of protein-coding data on 78 genes from 360 taxa, exploring the effects of different partitioning and character-coding protocols for the entire data set as well as subsets of the data. While our analyses recover many well-supported relationships and reveal strong support for some contentious relationships, several factors, including base composition biases, can affect the results. We also highlight the challenges of using plastid genome data in deep-level phylogenomic analyses and provide suggestions for future analyses that will incorporate plastid genome data for thousands of species.

## Results

### Data set

We assembled plastid protein-coding sequences from 360 species (Additional file 1) for which complete or nearly complete plastid genome sequences were available on GenBank. Of the 360 species, there were 258 angiosperms (*Angiospermae*), 53 gymnosperms (*Acrogymnospermae*, including three *Gnetophyta*), seven monilophytes (*Monilophyta*), four lycophytes (*Lycopodiophyta*), three liverworts (*Marchantiophyta*), one hornwort (*Anthocerotophyta*), two mosses (*Bryophyta*), six taxa from the paraphyletic streptophytic algae, and 26 chlorophytic algae (*Chlorophyta*). The phylogenetic character matrices contained sequences from 78 genes and the following number of alignment positions: 58,347 bp for the matrix containing all nucleotide positions (ntAll) and the RY-coded (RY) version of the ntAll matrix; 38,898 bp in the matrix containing only the first and second codon positions (ntNo3rd), and 19,449 amino acids (AA). The number of genes present per taxon varied from 18 to 78 (mean = 70), while the number of taxa present per gene ranged from 228 to 356 (mean = 322; see Additional file 2). Taxa with few genes present, such as *Helicosporidium* (18 genes) and *Rhizanthella* (19 genes), represent highly modified complete plastid genomes of non-photosynthetic species [70,71]. The percentage of missing data (gaps and ambiguous characters) was ~15.6% for each of the four data sets. The pattern of data across each of the four matrices is decisive, meaning that it can uniquely define a single tree for all taxa [72]. The data contain 100% of all possible triplets of taxa, and are decisive for 100% of all possible trees. All alignments have been deposited in the Dryad Data Repository [73].

### GC bias

**Figure 1:**
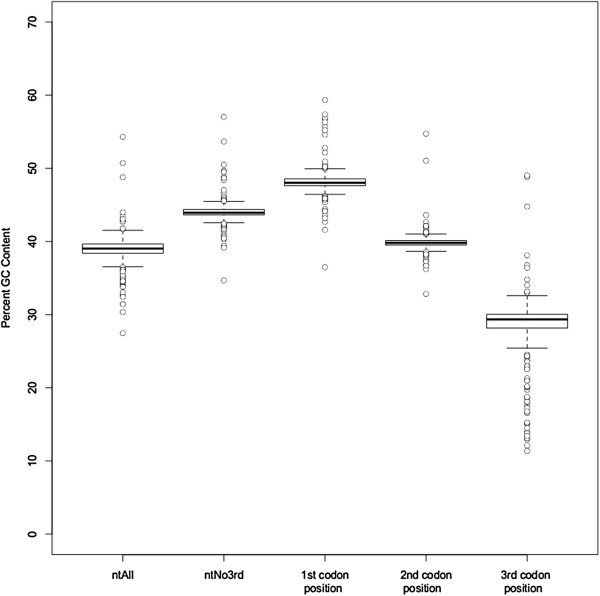
Box plots of percent GC content in the ntAll and ntNo3rd data sets as well as in the first, second, and third codon positions of the ntAll data set.

**Figure 2:**
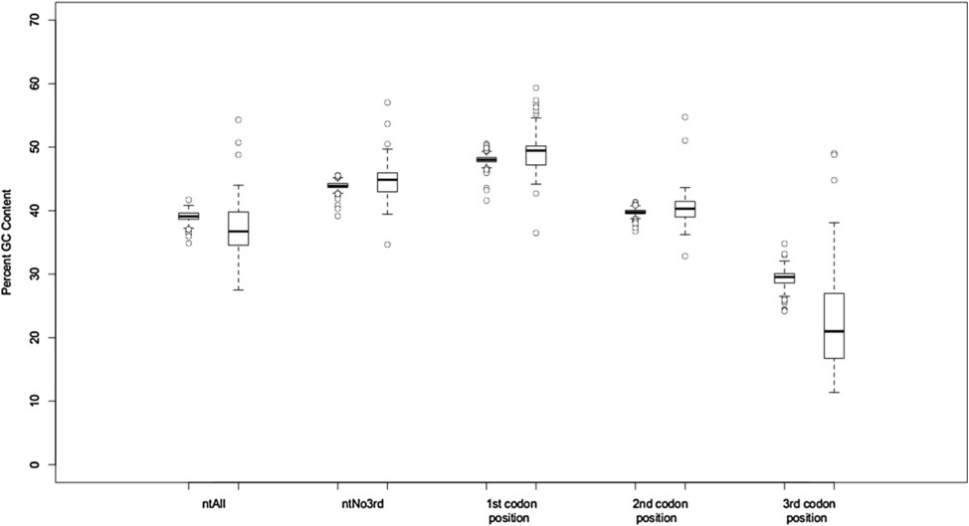
**Box plots of percent GC content in seed plants (*Spermatophyta; *on left) and the data set as a whole (*Viridiplantae; *on right) in the ntAll and ntNo3rd data sets as well as the first, second, and third codon positions of the ntAll data set.** For each pair of box plots, values for seed plants (*Spermatophyta*) are on the left, and values for all green plant taxa (*Viridiplantae*) are on the right.

**Figure 3:**
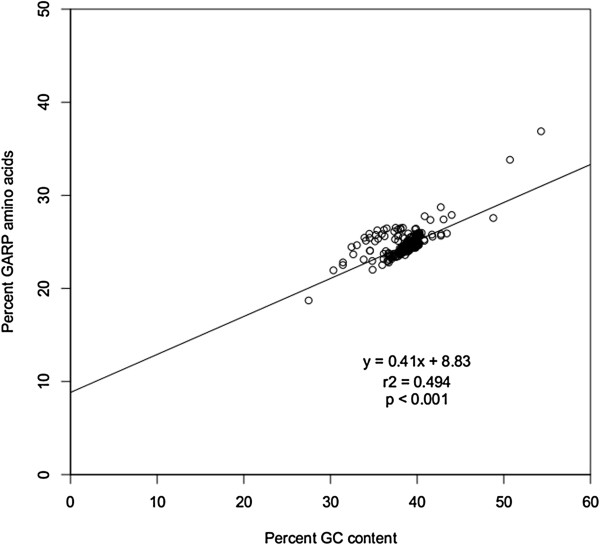
Correlation between percent GC nucleotide content in the ntAll matrix and percent of amino acids in the AA matrix that are coded for by GC-rich codons (G, A, R, and P).

**Figure 4:**
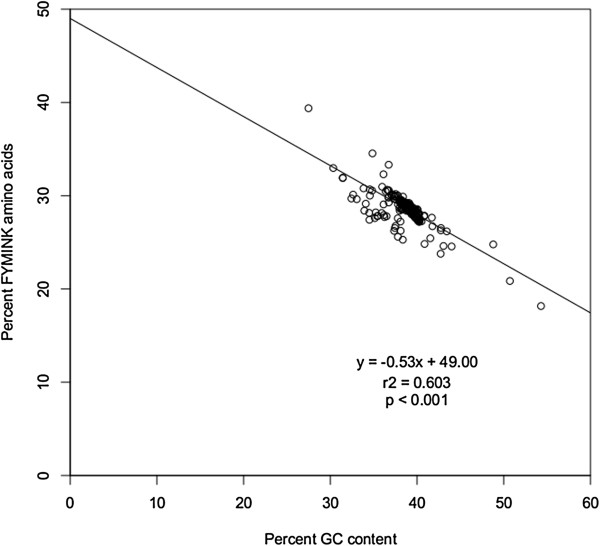
Correlation between percent GC nucleotide content in the ntAll matrix and percent of amino acids in the AA matrix that are coded for by AT-rich codons (F, Y, M, I, N, and K).

GC content varied considerably both among lineages and also within single genomes, and chi-square tests rejected the null hypothesis of homogeneous base frequencies (Table 1). The average GC content in the ntAll matrix was 38.9%, and it ranged from 54.3% in *Selaginella uncinata* to 27.5% in *Helicosporidium* sp. (Figure 1, Additional file 3). Also, the average GC content varied among first, second, and third codon positions, with by far the most variation among lineages at the third codon position (Figure 1, Additional file 3). Although there was extensive heterogeneity in GC content across all species, there was relatively little variation among the seed plant taxa (Figure 2). There also was significant correlation between nucleotide composition and amino acid composition. Plastid genomes that are GC-rich had a significantly higher percentage (Figure 3; p < 0.001) of amino acids that are encoded by GC-rich codons (i.e., G, A, R, and P). Similarly, GC-rich plastid genomes had a significantly lower percentage (Figure 4; p < 0.001) of amino acids that are coded by AT-rich codons (i.e., F, Y, M, I, N, and K).

**Table 1:** Chi-square tests of nucleotide composition homogeneity among lineages

**Data**	**χ**^**2**^	**df**	**p**
ntAll	31350.257185	1077	< 0.0001
ntNo3rd	11968.002464	1077	< 0.0001
ntAll (Position 1)	8366.331439	1077	< 0.0001
ntAll (Position 2)	6003.338041	1077	< 0.0001
ntAll (Position 3)	46288.248785	1077	< 0.0001

### Phylogenetic analyses

In the phylogenetic analyses of all data sets and partitioning schemes, the partitioning strategy with the most partitions consistently fit the data best based on the AICc (Table 2). These best-fit models partitioned the AA matrix by gene (78 partitions) and the nucleotide (ntAll, ntNo3rd) and RY matrices by codon position and gene (234 partitions). All a posteriori bootstopping analyses indicated that convergence of support values had been reached after 100 replicates, and thus our choice of 200 replicates was more than sufficient to obtain reliable bootstrap values.

**Table 2:** AICc scores for each of the phylogenetic matrix partitioning strategies

**Matrix**	**Number of characters**	**Partitioning strategy**	**Number of partitions**	**Log-likelihood**	**AICc**	**ΔAICc**
ntAll	58,347	OnePart	1	−3135739.544116	6272952.811161	114533.884536
		CodonPart	3	−3099273.099639	6200056.468462	41637.541838
		GenePart	78	−3120195.077316	6243312.241766	84893.315142
		**CodonGenePart**	**234**	**−3076219.426792**	**6158418.926624**	**0**
RY	58,347	OnePart	1	−1239354.453402	2480173.246480	21572.787069
		CodonPart	3	−1235533.368070	2472537.854401	13937.394990
		GenePart	78	−1234706.178899	2471197.311314	12596.851903
		**CodonGenePart**	**234**	**−1228081.159986**	**2458600.459411**	**0**
ntNo3rd	38,898	OnePart	1	−1387913.034830	2777313.721117	30326.016847
		CodonPart	2	−1385570.086154	2772645.570816	25657.866546
		GenePart	78	−1376158.263023	2755293.787916	8306.083646
		**CodonGenePart**	**156**	**−1371218.716450**	**2746987.704270**	**0**
AA	19,449	OnePart	1	−1418038.152084	2837614.101717	8353.616354
		**GenePart**	**78**	**−1413039.660496**	**2829260.485363**	**0**

Partitioning strategies judged to be the best by the AICc are in bold.

**Figure 5:**
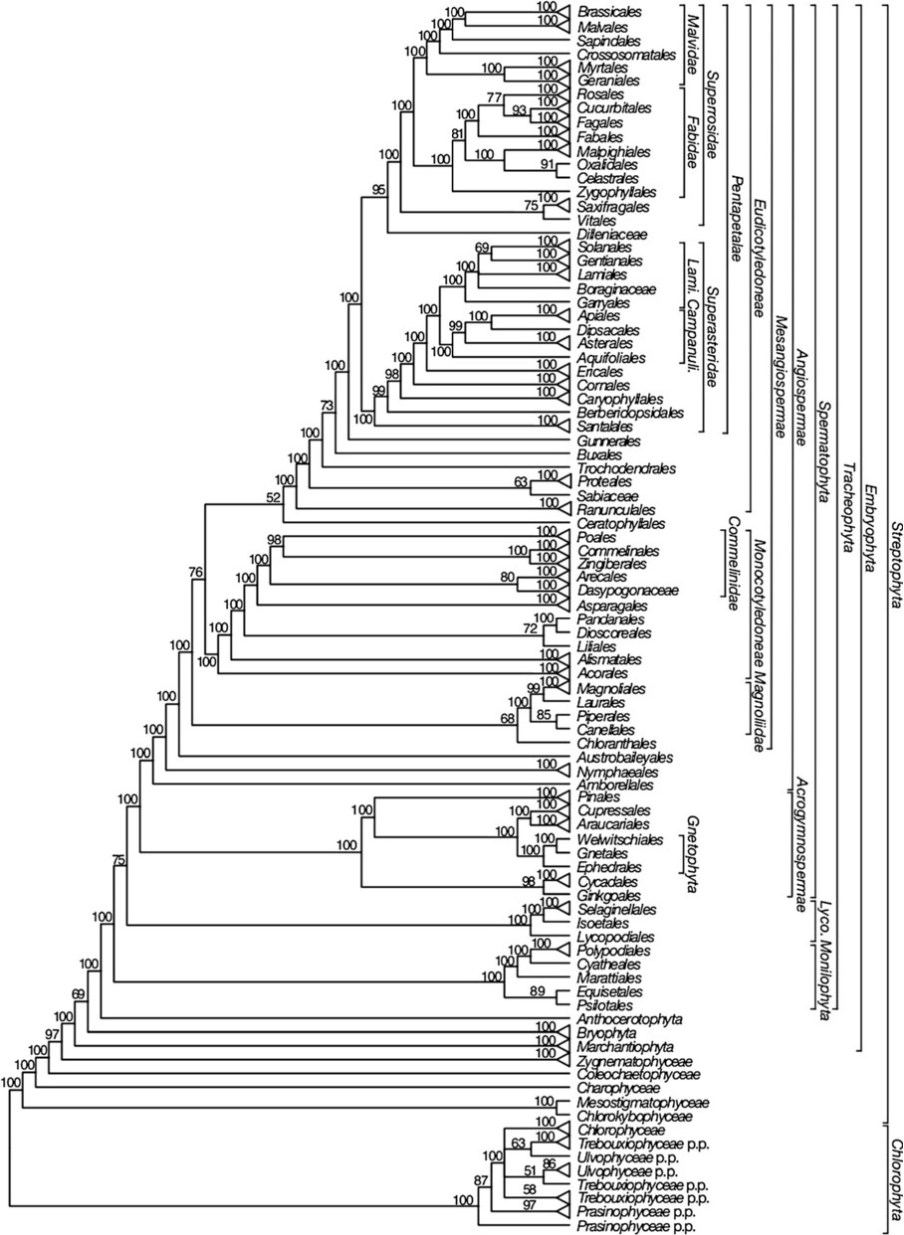
**Fifty percent maximum likelihood majority-rule bootstrap consensus summary tree of *Viridiplantae *inferred from the all nucleotide positions (ntAll) analysis.** Data set derived from 78 protein-coding genes of the plastid genome (ntax = 360; 58,347 bp; missing data ~15.6%). Bootstrap support values ≥ 50% are indicated. Terminals with a triangle represent collapsed clades with > 2 taxa. Note position of *Lycopodiophyta* as sister to *Spermatophyta* is likely caused by base composition bias (see text). See Figures 9, 10, 11, 12, 13, and 14 for the complete tree and Additional file 1 for taxonomy. *Lami*. = *Lamiidae*; *Campanuli*. = *Campanulidae*; *Lyco*. = *Lycopodiophyta*.

**Figure 6:**
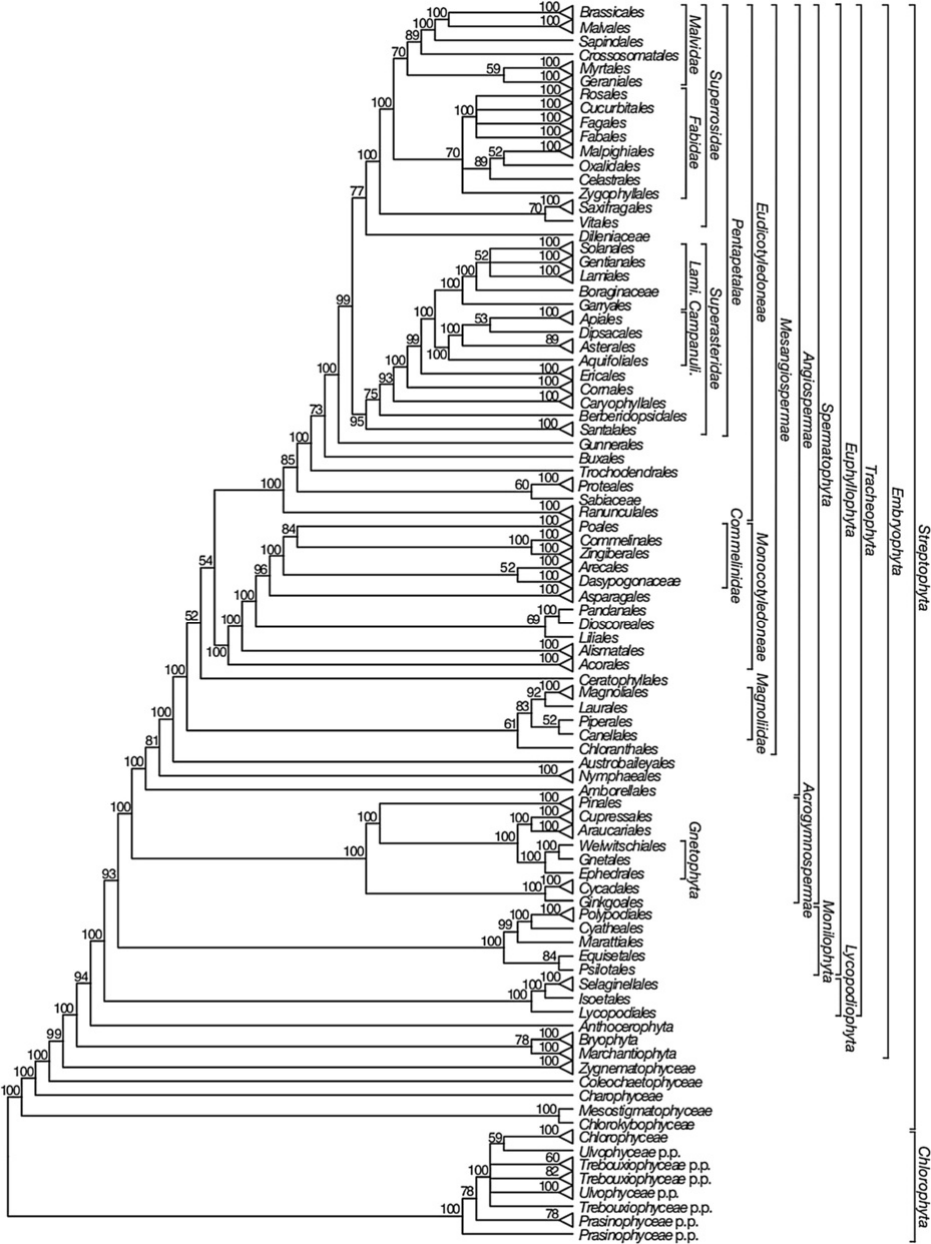
**Fifty percent maximum likelihood majority-rule bootstrap consensus summary tree of *Viridiplantae *inferred from the first and second codon positions (ntNo3rd) analysis.** Data set derived from 78 protein-coding genes of the plastid genome (ntax = 360; 38,898 bp; missing data ~15.6%). Bootstrap support values ≥ 50% are indicated. Terminals with a triangle represent collapsed clades with > 2 taxa. See Additional file 4 for the complete tree and Additional file 1 for taxonomy. *Lami*. = *Lamiidae*; *Campanuli*. = *Campanulidae*.

**Figure 7:**
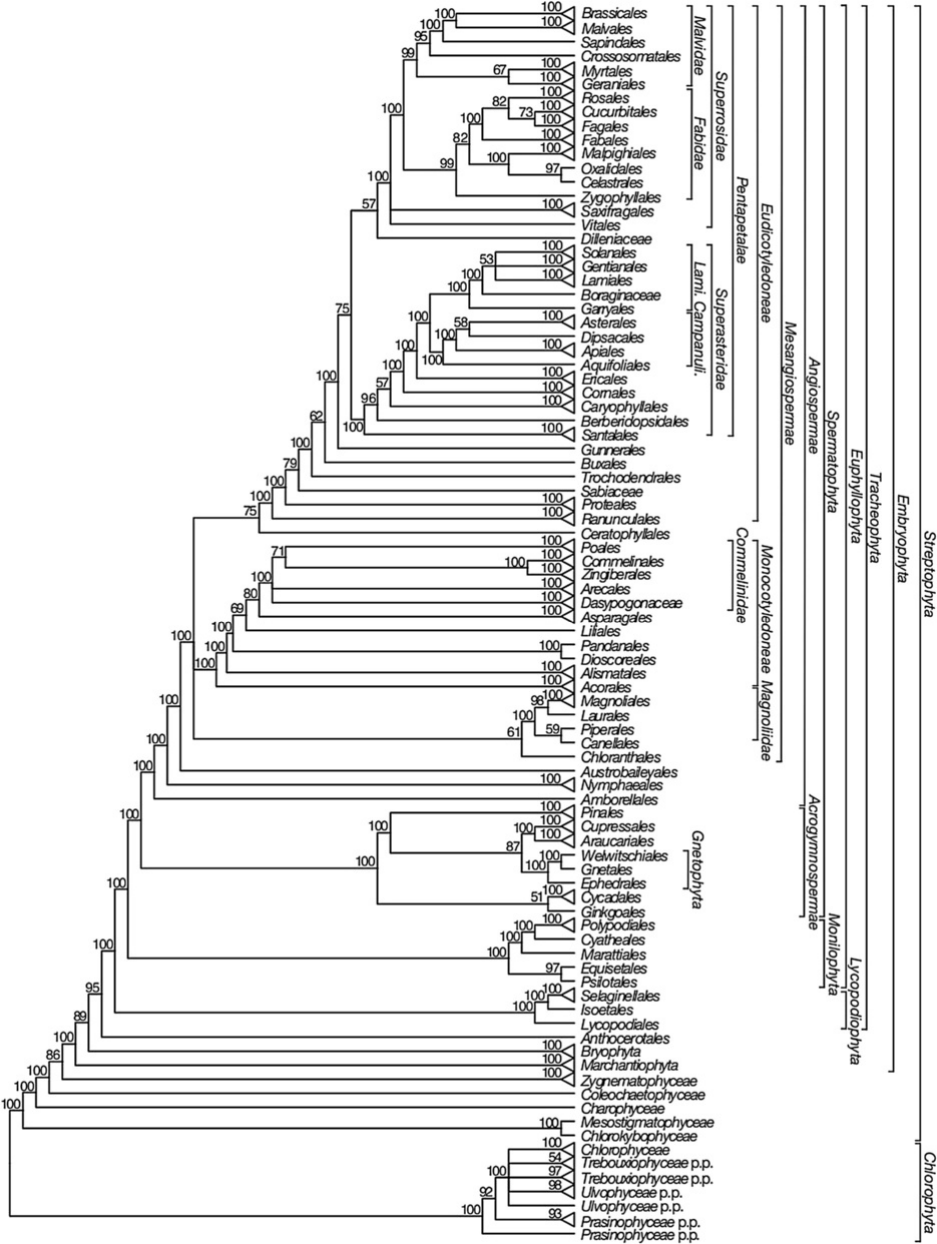
**Fifty percent maximum likelihood majority-rule bootstrap consensus summary tree of *Viridiplantae *inferred from the RY-coded (RY) analysis.** Data set derived from 78 protein-coding genes of the plastid genome (ntax = 360; 58,347 bp; missing data ~15.6%). Bootstrap support values ≥ 50% are indicated. Terminals with a triangle represent collapsed clades with > 2 taxa. See Additional file 5 for the complete tree and Additional file 1 for taxonomy. *Lami*. = *Lamiidae*; *Campanuli*. = *Campanulidae*.

**Figure 8:**
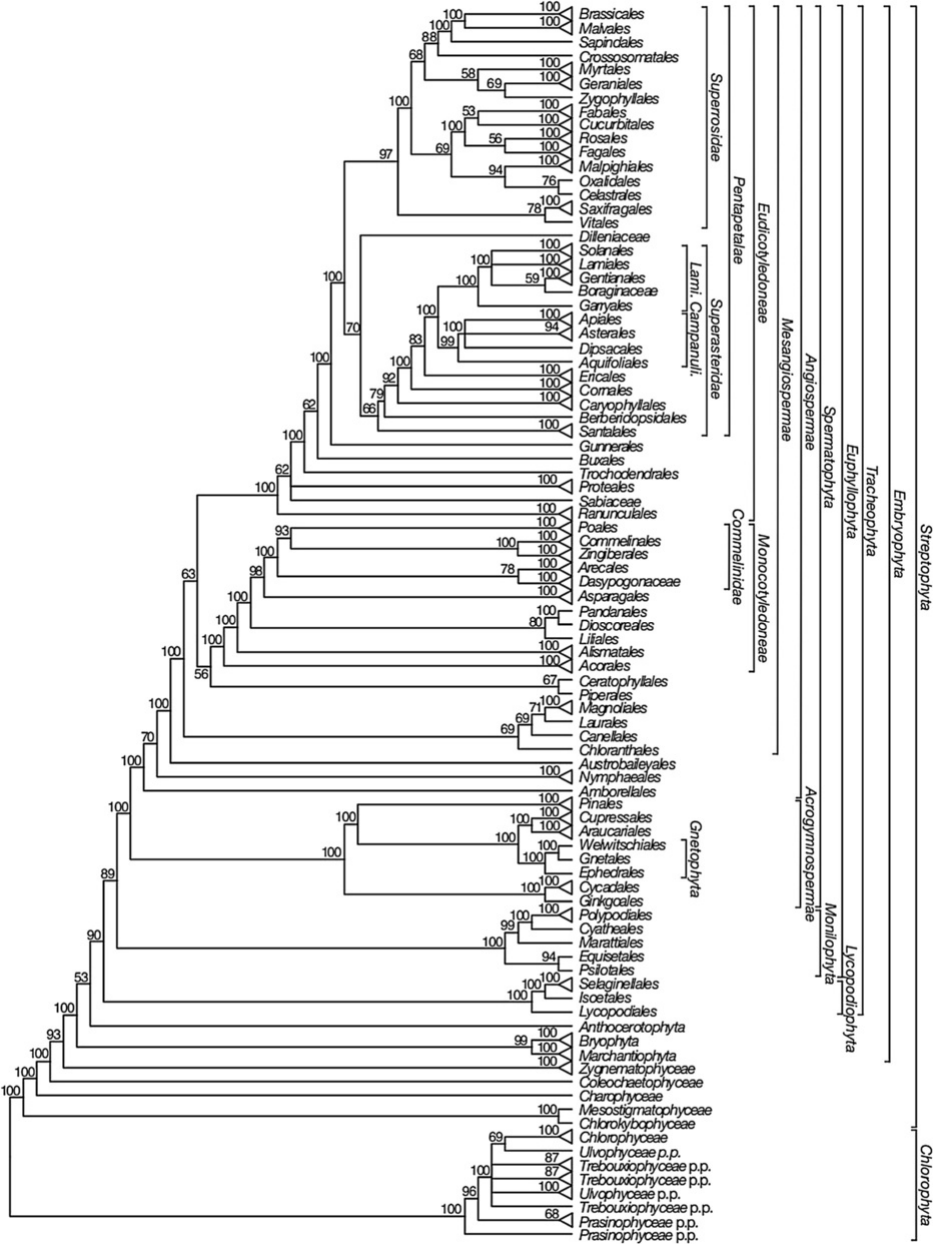
**Fifty percent maximum likelihood majority-rule bootstrap consensus summary tree of *Viridiplantae *inferred from the amino acid (AA) analysis.** Data set derived from 78 protein-coding genes of the plastid genome (ntax = 360; 19,449 AAs; missing data ~15.6%). Bootstrap support values ≥ 50% are indicated. Terminals with a triangle represent collapsed clades with > 2 taxa. See Additional file 6 for the complete tree and Additional file 1 for taxonomy. *Lami*. = *Lamiidae*; *Campanuli*. = *Campanulidae*.

**Figure 9:**
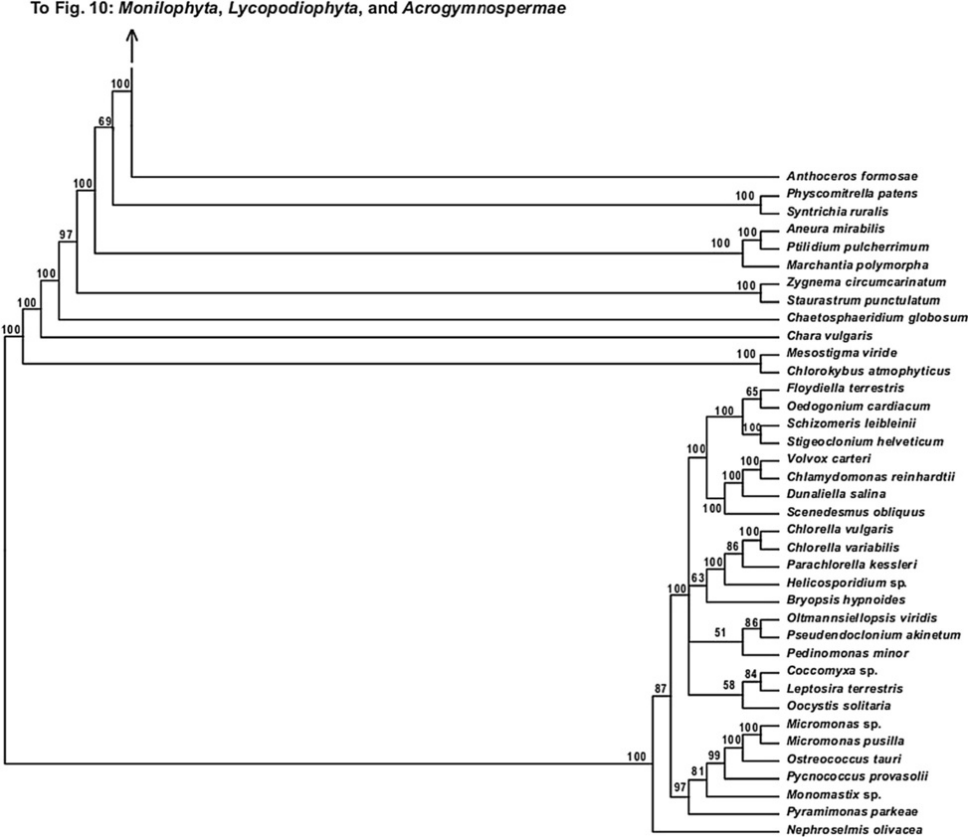
**Fifty percent maximum likelihood majority-rule bootstrap consensus tree of *Viridiplantae *inferred from the all nucleotide positions (ntAll) analysis. Portion of tree showing *Chlorophyta, Chlorokybophyceae, Mesostigmatophyceae, Charophyceae, Coleochaetophyceae, Zygnematophyceae, Marchantiophyta, Bryophyta, *and *Anthocerotophyta.*** Data set derived from 78 protein-coding genes of the plastid genome (ntax = 360; 58,347 bp; missing data ~15.6%). Bootstrap support values ≥ 50% are indicated. See also Figure 5 for a summary tree of major *Viridiplantae* clades and Additional file 1 for taxonomy. Tree continued in Figure 10.

**Figure 10:**
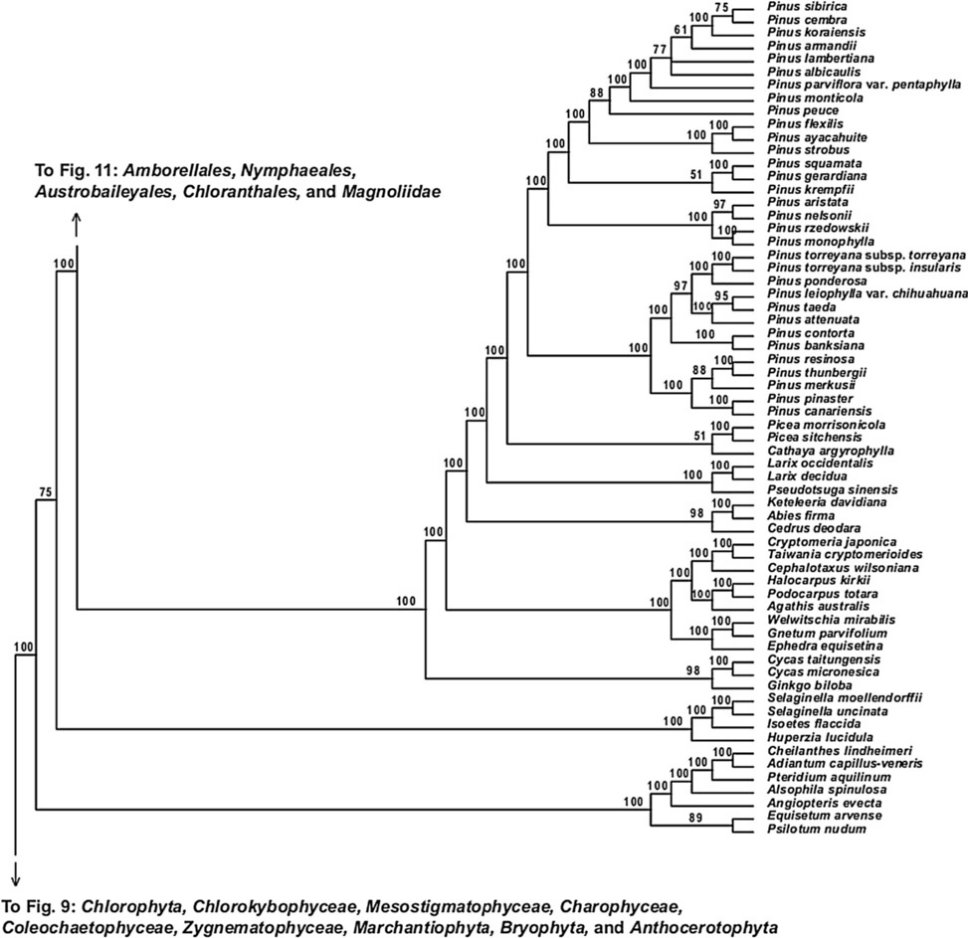
**Fifty percent maximum likelihood majority-rule bootstrap consensus tree of *Viridiplantae *inferred from the all nucleotide positions (ntAll) analysis. Portion of tree showing *Monilophyta***, ***Lycopodiophyta*, and *Acrogymnospermae.*** Data set derived from 78 protein-coding genes of the plastid genome (ntax = 360; 58,347 bp; missing data ~15.6%). Bootstrap support values ≥ 50% are indicated. See also Figure 5 for a summary tree of major *Viridiplantae* clades and Additional file 1 for taxonomy. Note position of *Lycopodiophyta* as sister to *Spermatophyta* is likely caused by base composition bias (see text). Tree continued in Figures 9 and 11.

**Figure 11:**
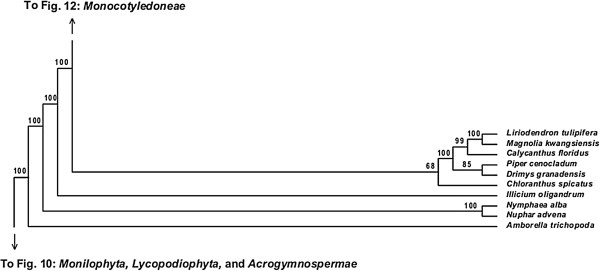
**Fifty percent maximum likelihood majority-rule bootstrap consensus tree of *Viridiplantae *inferred from the all nucleotide positions (ntAll) analysis. Portion of tree showing *Amborellales, Nymphaeales, Austrobaileyales, Chloranthales, *and *Magnoliidae.*** Data set derived from 78 protein-coding genes of the plastid genome (ntax = 360; 58,347 bp; missing data ~15.6%). Bootstrap support values ≥ 50% are indicated. See also Figure 5 for a summary tree of major *Viridiplantae* clades and Additional file 1 for taxonomy. Tree continued in Figures 10 and 12.

**Figure 12:**
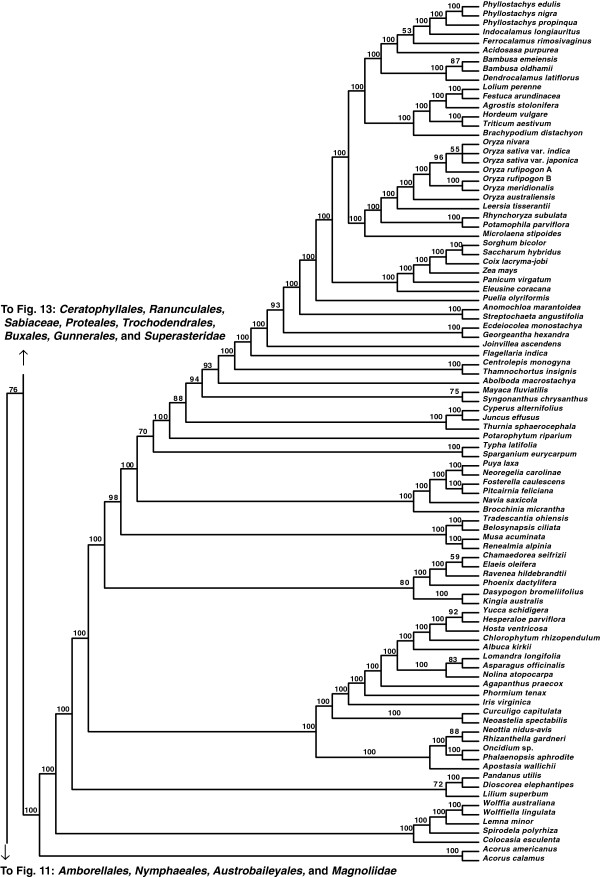
**Fifty percent maximum likelihood majority-rule bootstrap consensus tree of *Viridiplantae *inferred from the all nucleotide positions (ntAll) analysis. Portion of tree showing *Monocotyledoneae.*** Data set derived from 78 protein-coding genes of the plastid genome (ntax = 360; 58,347 bp; missing data ~15.6%). Bootstrap support values ≥ 50% are indicated. See also Figure 5 for a summary tree of major *Viridiplantae* clades and Additional file 1 for taxonomy. Tree continued in Figures 11 and 13.

**Figure 13:**
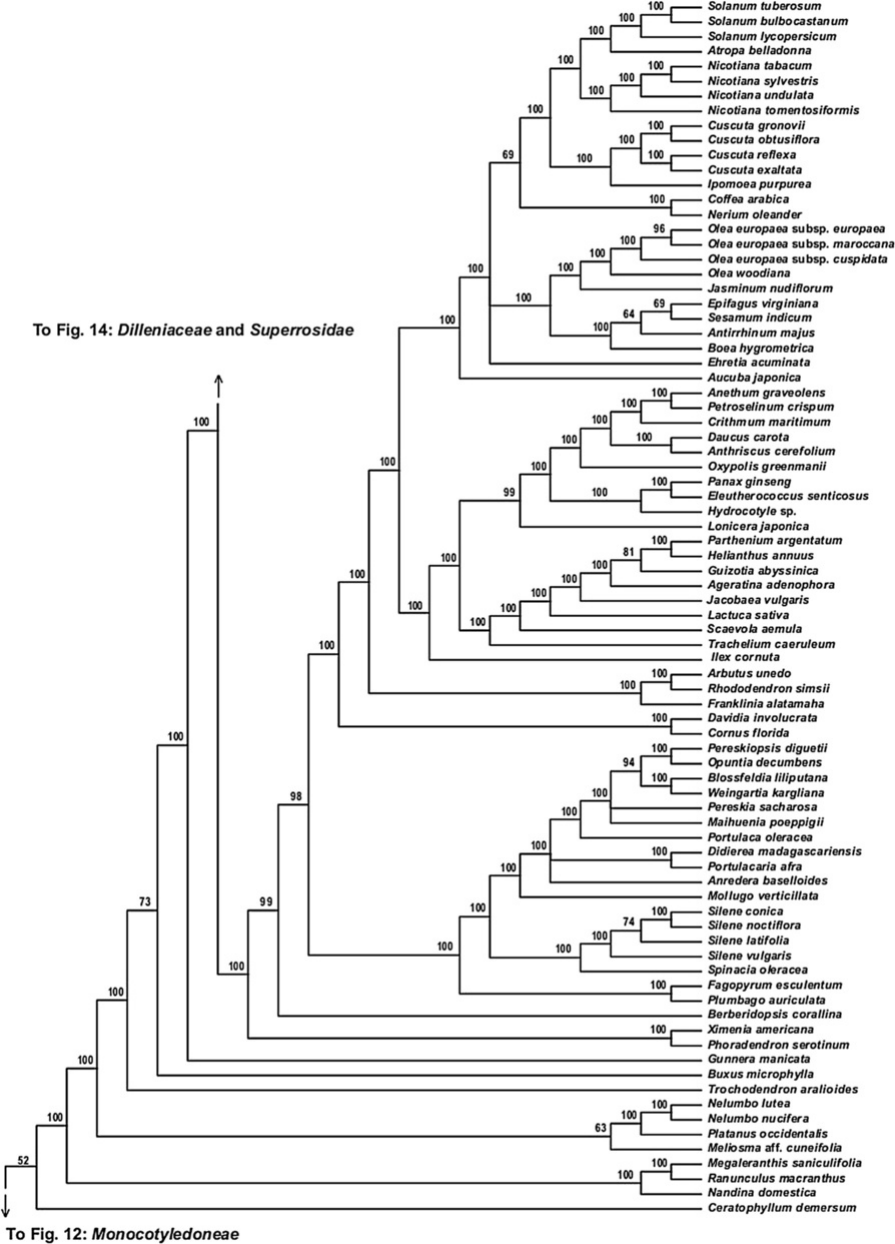
**Fifty percent maximum likelihood majority-rule bootstrap consensus tree of *Viridiplantae *inferred from the all nucleotide positions (ntAll) analysis. Portion of tree showing *Ceratophyllales, Ranunculales, Sabiaceae, Proteales, Trochodendrales, Buxales, Gunnerales, *and *Superasteridae.*** Data set derived from 78 protein-coding genes of the plastid genome (ntax = 360; 58,347 bp; missing data ~15.6%). Bootstrap support values ≥ 50% are indicated. See also Figure 5 for a summary tree of major *Viridiplantae* clades and Additional file 1 for taxonomy. Tree continued in Figures 12 and 14.

**Figure 14:**
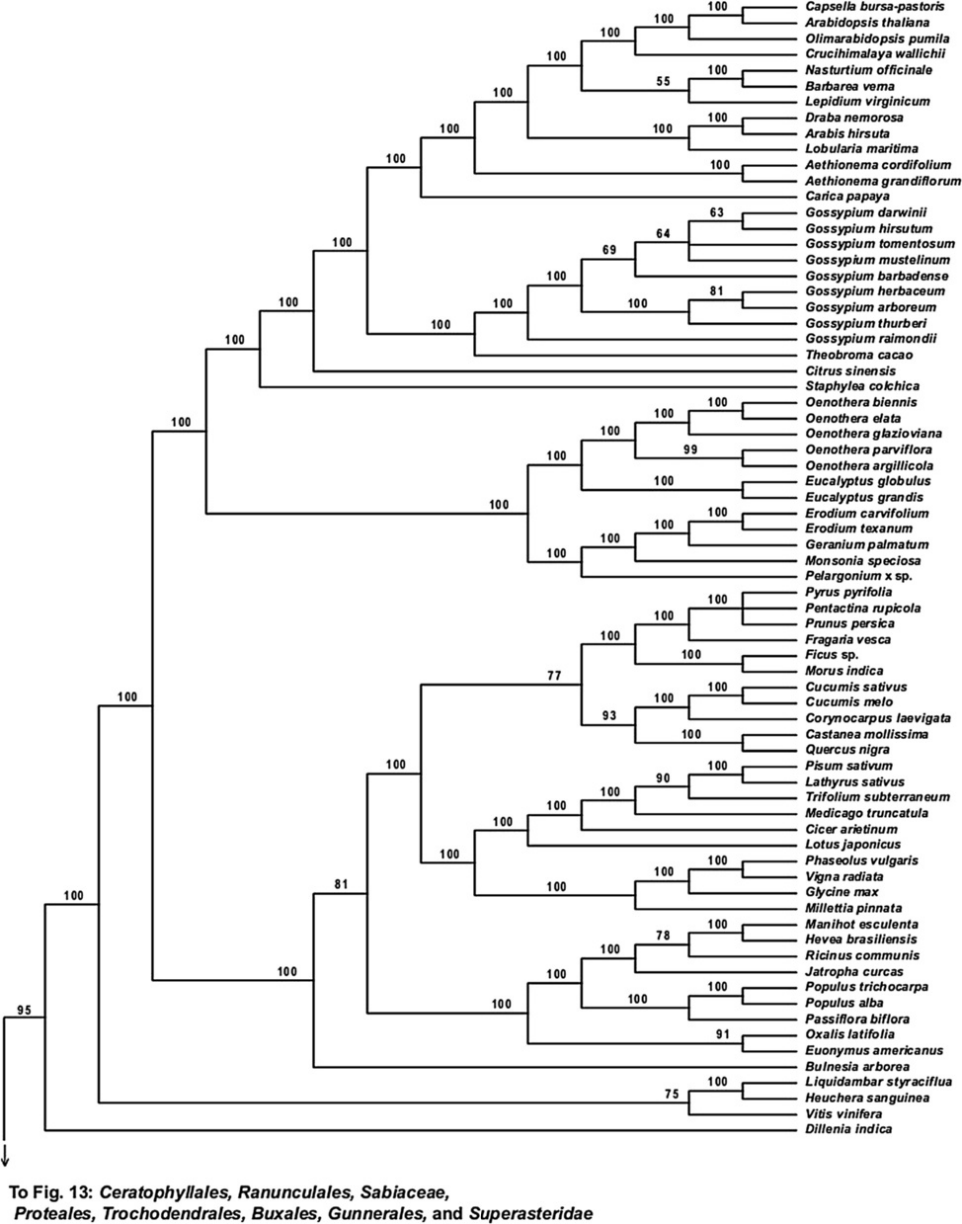
**Fifty percent maximum likelihood majority-rule bootstrap consensus tree of *Viridiplantae *inferred from the all nucleotide positions (ntAll) analysis. Portion of tree showing *Dilleniaceae *and *Superrosidae.*** Data set derived from 78 protein-coding genes of the plastid genome (ntax = 360; 58,347 bp; missing data ~15.6%). Bootstrap support values ≥ 50% are indicated. See also Figure 5 for a summary tree of major *Viridiplantae* clades and Additional file 1 for taxonomy. Tree continued in Figure 13.

We will focus on reporting the relationships of major clades of *Viridiplantae* shown in the 50% maximum likelihood (ML) majority-rule bootstrap consensus summary trees for each data set: ntAll (Figure 5), ntNo3rd (Figure 6), RY (Figure 7), and AA (Figure 8). These summary trees collapse some clades for ease of viewing the major relationships within *Viridiplantae*. A summary of important results and conflicts among these four data sets is given in Table 3. We provide full majority-rule bootstrap consensus trees for the ntAll (Figures 9, 10, 11, 12, 13, and 14), ntNo3rd (Additional file 4), RY (Additional file 5), and AA (Additional file 6) data sets. ML trees with branch lengths and BS values are also provided: ntAll (Additional file 7), ntNo3rd (Additional file 8), RY (Additional file 9), and AA (Additional file 10). Average support values among all internal nodes in the ML trees were slightly higher in the ntAll phylogeny (~94% bootstrap support [BS]; Additional file 7) compared to the other data sets (~90-91% BS; Additional files 8, 9, and 10). The ntAll phylogeny also had the most clades resolved with ≥ 70% BS (92%; 327 bipartitions resolved out of 357 possible) while the ntNo3rd, RY, and AA data sets had 87%, 87%, and 86% of the possible bipartitions resolved at ≥ 70% BS, respectively. All resulting trees have been deposited in the Dryad Data Repository [73].

**Table 3:** Summary of selected similarities and conflicts between bootstrap consensus topologies derived from the four data sets

**Taxon**	**ntAll**	**ntNo3rd**	**RY**	**AA**
*Amborellales*	sister to all other *Angiospermae* (100%/100%)	sister to all other *Angiospermae* (100%/81% )	sister to all other *Angiospermae* (100%/100%)	sister to all other *Angiospermae* (100%/70%)
*Anthocerotophyta*	sister to *Tracheophyta* (100%/100%)	sister to *Tracheophyta* (94%/100%)	sister to *Tracheophyta* (95%/100%)	sister to *Tracheophyta* (53%/90%)
*Ceratophyllales*	sister to *Eudicotyledoneae* (52%/100%)	sister to *Monocotyledoneae* + *Eudicotyledoneae* (52%/54%)	sister to *Eudicotyledoneae* (75%/100%)	sister to *Piperales* (67%)
COM clade	within *Fabidae* (100%)	within *Fabidae* (70%)	within *Fabidae* (99%)	sister to a clade including *Cucurbitales, Rosales, Fabales, Fagales* (69%/100%; *Fabidae* not monophyletic)
*Dilleniales*	sister to *Superrosidae* (95%/100%)	sister to *Superrosidae* (77%/100%)	sister to *Superrosidae* (57%/100%)	sister to *Superasteridae* (70%/66%)
*Ginkgoales*	sister to *Cycadales* (98%/100%)	sister to *Cycadales* (100%/100%)	sister to *Cycadales* (51%/100%)	sister to *Cycadales* (100%/100%)
*Gnetophyta*	sister to *Cupressales* + *Araucariales* (100%/100%)	sister to *Cupressales* + *Araucariales* (100%/100%)	sister to *Cupressales* + *Araucariales* (87%/100%)	sister to *Cupressales* + *Araucariales* (100%/100%)
*Marchantiophyta*	sister to all other *Embryophyta* (100%/69%)	sister to *Bryophyta* (78%/100%)	sister to all other *Embryophyta* (100%/89%)	sister to *Bryophyta* (99%/100%)
*Monilophyta*	sister to *Lycopodiophyta* + *Spermatophyta* (100%/75%)	sister to *Spermatophyta* (93%/100%)	sister to *Spermatophyta* (100%/100%)	sister to *Spermatophyta* (89%/100%)
*Prasinophyceae*	not monophyletic; *Nephroselmis* sister to all other *Chlorophyta* (100%/87%)	not monophyletic; *Nephroselmis* sister to all other *Chlorophyta* (100%/78%)	not monophyletic; *Nephroselmis* sister to all other *Chlorophyta* (100%/92%)	not monophyletic; *Nephroselmis* sister to all other *Chlorophyta* (100%/96%)
*Zygnematophyceae*	sister to *Embryophyta* (97%/100%)	sister to *Embryophyta* (99%/100%)	sister to *Embryophyta* (86%/100%)	sister to *Embryophyta* (93%/100%)

Bootstrap support (BS) values >50% are shown as percentages. When sister groups for the taxon of interest are listed, bootstrap support (BS) values on the left are for the clade including the taxon of interest and its sister group within *Viridiplantae,* while BS values on the right are for the more inclusive clade excluding the taxon of interest. If only one BS value is given for a sister relationship, only two terminals are involved (see also Figures 5, 6, 7, and 8).

The monophyly of *Chlorophyta* receives 100% BS in all analyses. *Prasinophyceae* are consistently not monophyletic. Instead, the prasinophyte *Nephroselmis* is sister to all other *Chlorophyta* (Figure 9; Additional files 4, 5, and 6), while remaining *Prasinophyceae* form a clade that is variously supported (ntAll 97% BS, ntNo3rd 78% BS, RY 93% BS, and AA 68% BS) and is sister to a clade of the remaining *Chlorophyta. Chlorophyceae* are monophyletic (100% BS in all analyses), but *Trebouxiophyceae* and *Ulvophyceae* are not monophyletic, and the relationship of *Chlorophyceae* to these lineages is unresolved.

We consistently recovered a single set of relationships among the streptophytic algae subtending the land plant clade. *Zygnematophyceae* are sister to land plants, *Coleochaetophyceae* are sister to *Zygnematophyceae* + *Embryophyta*, *Charophyceae* are sister to *Coleochaetophyceae* + (*Zygnematophyceae* + *Embryophyta*), and a clade of *Mesostigmatophyceae* + *Chlorokybophyceae* is sister to all other *Streptophyta*. Each of these relationships has ≥86% BS support (Figures 5, 6, 7, and 8).

The branching order of the non-vascular land plant lineages differs among analyses. In analyses of the ntAll and RY data sets, *Marchantiophyta* (liverworts), followed by *Bryophyta* (mosses), and then *Anthocerotophyta* (hornworts) are the earliest-branching land plant lineages, with *Anthocerotophyta* the immediate sister to the vascular plants (*Tracheophyta*; Figures 5 and 7). In the ntAll and RY analyses, these relationships had ≥89% BS support except for the *Bryophyta* + (*Anthocerophyta* + *Tracheophyta*) relationship in the ntAll analysis, which received only 69% BS (Figure 5). In contrast, in the ntNo3rd and AA analyses, *Bryophyta* and *Marchantiophyta* formed a clade (78% BS [Figure 6] and 99% BS [Figure 8], respectively), followed by *Anthocerophyta* as sister to *Tracheophyta* (94% [Figure 6] and 53% BS [Figure 8], respectively).

Within *Tracheophyta*, the ntNo3rd, RY, and AA data sets all place *Lycopodiophyta* sister to a *Euphyllophyta* clade (*Monilophyta* + *Spermatophyta*; ≥89% BS, Figures 6, 7, and 8). However, the analysis of the ntAll data set places *Monilophyta* sister to a clade of *Lycopodiophyta* + *Spermatophyta* (75% BS, Figures 5, 6, 7, 8, 9, and 10).

Our analyses of *Monilophyta* generally reveal strong support for a clade of *Equisetales* + *Psilotales* as sister to *Marattiales* + leptosporangiate ferns (represented by *Cyatheales* and *Polypodiales*). The lowest support obtained was for *Equisetales* + *Psilotales* in the ntNo3rd analysis (84% BS; Figure 6) and ntAll (89% BS; Figure 5); all other nodes in all analyses received > 90% BS, with *Marattiales* + leptosporangiate ferns receiving ≥ 99% BS.

Within *Spermatophyta*, all analyses place the extant gymnosperms (*Acrogymnospermae*) sister to *Angiospermae* with 100% BS. Within extant gymnosperms, *Cycadales* and *Ginkgoales* form a clade (≥ 98% BS in ntAll, ntNo3rd, and AA; 51% BS in RY) that is sister to a clade in which *Gnetophyta* (100% BS in all analyses) are nested within the paraphyletic conifers. There is generally high support (100% BS in ntAll [Figure 5], ntNo3rd [Figure 6], and AA [Figure 7]; 87% BS [Figure 8] in RY) placing *Gnetophyta* as sister to a clade of *Araucariales* + *Cupressales*. This “Gnecup” clade [sensu 16, 30, 41] is then sister to *Pinales,* which has 100% BS in all analyses.

In all analyses, *Angiospermae* receive 100% BS, and *Amborella* (*Amborellales*) is sister to all other angiosperms, followed by *Nymphaeales*, and then *Austrobaileyales*. These relationships are mostly supported by 100% BS. However, *Nymphaeales* + (*Austrobaileyales* + *Mesangiospermae*) receives 81% BS (Figure 6) in the ntNo3rd analyses and 70% BS (Figure 8) in the AA analyses. The remaining angiosperms (*Mesangiospermae*) receive 100% BS in all analyses. Within *Mesangiospermae,* the relationships among *Monocotyledoneae*, *Magnoliidae*, *Eudicotyledoneae*, and *Ceratophyllum* (*Ceratophyllales*) are not well supported and vary depending on the analysis. The strongest support for the placement of *Ceratophyllales* is 75% BS as sister to *Eudicotyledoneae* in the RY analysis (Figure 7).

*Chloranthales* receive 61-69% BS as sister to the well-supported (100% BS in ntAll, RY; 83% BS in ntNo3rd) *Magnoliidae*. However, *Magnoliidae* are not monophyletic in the AA analyses, where *Piperales* are sister to *Ceratophyllales* (67% BS; Figure 8).

Within the monocot clade (*Monocotyledoneae*), *Acorales*, followed by *Alismatales*, have 100% BS in all analyses as subsequent sisters to the remaining monocots. In three of our analyses (ntAll, ntNo3rd, and AA), a variously supported clade (72%, 69%, and 80% BS, respectively) of *Liliales* + (*Pandanales* + *Dioscoreales*) is sister to a clade (>95% BS in these three analyses) of the remaining monocots (*Asparagales* + *Commelinidae*). However, in the RY-coded analysis, *Pandanales* + *Dioscoreales* (100% BS) is sister to a clade of *Liliales* + (*Asparagales* + *Commelinidae*), which receives 69% BS (Figure 7). Here *Asparagales* + *Commelinidae* is supported by 80% BS.

Within the eudicots (*Eudicotyledoneae*), which receive 100% BS in all analyses, *Ranunculales* are sister to the remaining taxa. In the ntAll, ntNo3rd, RY, and AA analyses, the clade of these remaining taxa receives 100%, 85%, 100%, and 62% BS, respectively. Relationships vary among *Sabiaceae*, *Proteales*, and a clade of the remaining taxa, depending on the analysis. In the ntAll and ntNo3rd analyses, *Proteales* + *Sabiaceae* are supported as a clade, although with only 63% and 60% BS, respectively. However, in the RY analysis, *Proteales* are sister to a clade containing *Sabiaceae* plus the remaining taxa, which has 79% BS. In the AA analysis, relationships among these three clades are unresolved.

Among the remaining eudicots, we consistently recovered *Trochodendrales* as sister to *Buxales* + *Pentapetalae* and *Gunnerales* as sister to the remaining lineages of *Pentapetalae*: *Dilleniaceae*, *Superrosidae*, and *Superasteridae*. The placement of *Dilleniaceae* remains uncertain. The family is sister to *Superrosidae* in the ntAll (95% BS), ntNo3rd (77% BS), and RY (57% BS) analyses, but appears as sister to *Superasteridae* (70% BS) in the AA analysis.

Within *Superrosidae*, a clade of *Vitales* + *Saxifragales* is supported in the ntAll (75% BS), ntNo3rd (70% BS), and AA (78% BS) analyses. In the RY analysis, the relationship among *Saxifragales*, *Vitales*, and remaining *Rosidae* (*Fabidae* + *Malvidae*) is unresolved. *Fabidae* and *Malvidae* are both recovered with ≥ 99% BS in the ntAll and RY analyses. However, each clade receives only 70% BS in the ntNo3rd analysis. In the AA analysis neither clade is monophyletic; *Zygophyllales* are embedded (68% BS) within a clade of *Malvidae* taxa. The COM clade (*Celastrales*, *Oxalidales*, *Malpighiales*) is sister to a clade of *Fagales*, *Cucurbitales*, *Rosales*, and *Fabales* in *Fabidae* in the AA (69% BS; Figure 8), RY (82% BS; Figure 7), and ntAll (81% BS; Figure 5) trees and forms a trichotomy with *Zygophyllales* and the clade of *Fagales*, *Cucurbitales*, *Rosales*, and *Fabales* in the ntNo3rd tree (70% BS; Figure 6). *Zygophyllales* are sister to *Geraniales* (69% BS; Figure 8) in the AA tree and sister to all other *Fabidae* in the ntAll and RY trees (with 100% [Figure 5] and 99% BS [Figure 7], respectively).

*Superasteridae* (*Santalales*, *Berberidopsidales*, *Caryophyllales*, and *Asteridae*) are recovered in all analyses. This clade receives 100% BS in the ntAll and RY analyses, 95% BS in the ntNo3rd analysis, and 66% BS in the AA analysis. *Santalales* and *Berberidopsidales* are strongly supported as subsequent sisters to *Caryophyllales* + *Asteridae*. Within *Asteridae*, *Cornales*, followed by *Ericales*, are subsequent sisters to a strongly supported clade that comprises strongly supported *Campanulidae* and *Lamiidae* clades. Within *Lamiidae*, the placement of *Boraginaceae* is weak among the various analyses. *Boraginaceae* are sister to *Gentianales* (59% BS; Figure 8) in the AA tree, part of a trichotomy (100% BS; Figure 5) with *Lamiales* and *Solanales* + *Gentianales* in the ntAll tree, and sister to a weakly supported clade including *Gentianales*, *Lamiales*, and *Solanales* in the ntNo3rd (Figure 6) and RY (Figure 7) trees.

Analysis of only the third codon positions (nt3rdOnly, Additional file 11) resulted in several very strong conflicts along the backbone of *Viridiplantae* when compared to the topology from the ntNo3rd analyses. These conflicts include the backbone relationships within *Chlorophyta*, the placements of *Cycadales* and *Lycopodiophyta*, the relationships of the three major bryophyte lineages, and backbone relationships within *Poales*. Removal of four taxa (*Epifagus, Helicosporidium, Neottia,* and *Rhizanthella)* with elevated rates of molecular evolution and few genes present in the data sets did not significantly affect the resulting topologies.

## Discussion

While the enormous phylogenetic data sets that result from new genome or transcriptome sequencing efforts can ameliorate the effects of random or stochastic error, they also may exacerbate the effects of systematic error, or error resulting from problems in the analysis, such as model inaccuracy. The high amount of agreement among our various analyses and strong support for results generally consistent with previous studies (many of which also used plastid genes) suggest that plastid genome sequence data hold much promise for resolving relationships throughout the green plants. However, several areas of conflict between analyses using different character-coding strategies demonstrate that plastid genome phylogenetics is also susceptible to systematic error. Here we evaluate the phylogenetic results, emphasizing areas of agreement and concern, and then address some of the methodological issues raised by our results.

### Evaluation of phylogenetic relationships

Historically, *Chlorophyta* have been divided into *Prasinophyceae*, *Trebouxiophyceae*, *Chlorophyceae*, and *Ulvophyceae* based on the ultrastructure of the flagellar apparatus and features related to cytokinesis [74,75]. The current status of green algae phylogenetics (*Chlorophyta* and streptophytic algae) has been reviewed recently [26,76,77]. The most comparable study to ours in terms of data and taxon sampling is by Lang and Nedelcu [26], who constructed a phylogeny of green algae with plastid genome sequence data. However, they analyzed only an amino acid data set using Bayesian inference and the CAT model [78,79]. We found a paraphyletic *Prasinophyceae* (not including *Pedinomnas*; Figures 5, 6, 7 and 8), which agrees with previous molecular analyses [26,76,77]. However, Lang and Nedelcu [26] recovered a monophyletic *Prasinophyceae*, albeit with little support. *Chlorophyceae* are monophyletic (100% BS in all of our analyses), which agrees with the results of Lang and Nedelcu [26]. We also find that *Trebouxiophyceae* and *Ulvophyceae* are not monophyletic, and that the relationship of *Chlorophyceae* to these lineages is unresolved. The branching order of the various *Trebouxiophyceae*, *Ulvophyceae*, and *Chlorophyceae* lineages within *Chlorophyta*, unresolved in our analyses, was also uncertain in earlier analyses (reviewed in [26,76,77]). Similarly in Lang and Nedelcu [26], *Trebouxiophyceae* and *Ulvophyceae* were not supported as monophyletic, although unlike our results, almost all nodes in their phylogeny were maximally supported.

Our analyses provide consistent, strong support for the relationships of streptophytic algae to land plants, and all analyses support *Zygnematophyceae* as the sister to land plants (Figures 5, 6, 7, and 8). Relationships among these lineages and the closest relatives of land plants have varied in previous studies depending on taxon sampling and gene choice. Some studies agree with our results placing *Zygnematophyceae* as sister to land plants [25,27,80-82], while other phylogenetic analyses indicate that *Charophyceae*[23,83,84] or *Coleochaetophyceae*[26,40,85,86] occupy this position. Depending on the analysis, Zhong et al. [87] found either *Zygnematophyceae* alone or a clade of *Zygnematophyceae* + *Coleochaetophyceae* as sister to land plants. In particular, the results of Lang and Nedelcu [26] conflict with our results regarding the sister group to *Embryophyta*. While we find a clade of *Coleochaetophyceae* + (*Zygnematophyceae* + *Embryophyta*), their results strongly support *Zygnematophyceae* + (*Coleochaetophyceae* + *Embryophyta*).

Phylogenetic relationships among bryophytes (mosses, hornworts, and liverworts) are also contentious, and nearly every possible relationship among these lineages has been reported, often with strong support. Most studies have shown the bryophytes as paraphyletic with respect to *Tracheophyta* rather than as a clade [30-33]. As recovered in our ntAll and RY analyses (Figures 5 and 7), liverworts (*Marchantiophyta*) often are placed sister to all other land plants, followed by mosses (*Bryophyta*), and with hornworts (*Anthocerotophyta*) sister to *Tracheophyta*[29,34,47,50,88,89]. A sister relationship between mosses and liverworts, found in our ntNo3rd and AA analyses (Figures 6 and 8), was proposed previously based on morphological [90-93] and molecular data [27,30,94,95] and has been recovered with numerous nuclear genes (Wickett et al., in review). This relationship was also recovered in analyses of complete plastid genome data by Karol et al. [34] when divergent taxa (i.e., *Selaginella* spp.) were excluded from phylogenetic analyses and also by Wolf and Karol [35] when third positions were excluded.

Our results placing *Lycopodiophyta* sister to *Euphyllophyta* in all but the ntAll analysis agree with most molecular phylogenetic analyses [29,96,97]. This split is also supported by analyses of morphological characters in fossil [15] and extant taxa [98]. *Monilophyta* and *Spermatophyta* also possess a 30-kb inversion in the large single-copy region of the plastid genome not found in *Lycopodiophyta* and the three bryophyte clades [99]. In the ntAll analysis, *Euphyllophyta* are not monophyletic (Figure 5); *Lycopodiophyta*, rather than *Monilophyta*, are sister to *Spermatophyta*. This relationship has been reported previously [34]; however, it likely is a phylogenetic artifact, perhaps related to base composition bias (see below). The plastid genome of the lycophyte *Selaginella* has an especially high GC content [21], with *Selaginella unicata* having the highest GC content in our ntAll data set (54.3%; Figure 1).

In some previous studies, relationships among lineages of *Monilophyta* have not been well resolved or supported (e.g., [29,89,96-98]). As a result, the relationships among *Equisetales*, *Psilotales*, *Marattiales*, and leptosporangiate ferns are often represented as a polytomy (e.g., [35]). In contrast, most of our analyses recovered strong support for a clade of *Equisetales* + *Psilotales* as sister to *Marattiales* + leptosporangiate ferns (represented here by *Cyatheales* and *Polypodiales*). These relationships agree with recent studies of monilophyte relationships based on plastid genome sequence data [34,35], although support is stronger here. Unfortunately, *Ophioglossales*, which often appear as sister to *Psilotales*, lacked a sequenced plastome at the time of our analyses. However, plastid genome data for *Ophioglossales* have subsequently been published and analyzed in a phylogenetic context [100], with strong support for *Ophioglossales* as sister to *Psilotales* and weak support for this clade as sister to *Equisetales*. Results from that study with regard to *Marattiales* and leptosporangiate ferns agree with the relationships presented here.

Relationships among the lineages of extant seed plants, and especially the placement of *Gnetophyta*, have long been debated [38,39,43,51,89,101]. Gnecup trees, found in all of our analyses, were initially recovered by Nickrent et al. [30], and then more recently by Zhong et al. [41]. However, Zhong et al. [41] suggested that the support for Gnecup may be the result of long-branch attraction; by removing highly variable proteins, support for Gnecup decreased. Furthermore, by removing what they considered parallel substitutions between lineages leading to *Gnetophyta* and to *Cryptomeria* (the sole *Cupressales* in their analyses), a Gnepine topology was recovered. Although several different placements for *Gnetophyta* have been recovered and strongly supported, many studies involving multiple genes have placed *Gnetophyta* sister to *Pinales* (Gnepine; [38,39,43,89], Wickett et al., in review). Using both coalescent and concatenation analyses, Xi et al. [102] found that the phylogenetic placement of *Gnetophyta* differs between the nuclear and plastid genomes. In their analyses using nuclear data, the Gnepine hypothesis is supported, while their analyses of plastid data support the Gnecup hypothesis. In contrast, Lee et al. [46] found strong support for *Gnetophyta* sister to the remaining gymnosperms [(*Cycadales* + *Ginkgoales*) + conifers)] in an ML analysis of 22,833 sets of nuclear gene orthologs from 101 land plant genera.

The backbone relationships among angiosperm (*Angio-spermae*) lineages generally agree with results from recent analyses, including a 17-gene analysis of 632 angiosperms [103] and previous analyses of plastid genome data sets [63,104-106]. The position of *Ceratophyllum* (*Ceratophyllales*), and thus the relationships among *Monocotyledoneae*, *Eudicotyledoneae*, and *Magnoliidae,* varies among our analyses, although without strong support. This contrasts with several other large, multi-gene analyses in which *Monocotyledoneae* are sister to *Ceratophyllales* + *Eudicotyledoneae*[63,103,106]. Interestingly, the strongest support for the placement of *Ceratophyllales* sister to *Eudicotyledoneae* is in the RY analysis (75% BS; Figure 7). However, in that analysis, the relationships among *Ceratophyllales* + *Eudicotyledoneae*, *Monocotyledoneae*, and *Magnoliidae* are unresolved*.*

Within the angiosperms, some relationships that have been uncertain, particularly at deep levels (reviewed in [103,107]), receive moderate to strong support in at least some of our analyses. For example, the placement of *Myrtales* and *Geraniales* in the *Malvidae* is supported with 70% BS (Figure 6) in the ntNo3rd tree and ≥ 99% BS in the RY (Figure 7) and ntAll (Figure 5) trees. *Myrtales* and *Geraniales* are also placed in a clade with the *Malvidae* taxa in the AA analysis (68% BS; Figure 8); however, *Zygophyllales* are also included within this clade, making *Malvidae* non-monophyletic. Likewise, *Chloranthales* are sister to *Magnoliidae* in all trees, but with weaker support (61% BS for RY and ntNo3rd, 68% BS for ntAll, and 69% BS for AA, but with *Piperales* removed from *Magnoliidae* in the latter). In two cases, all analyses but RY resolve relationships (although often with only moderate support), with RY producing a polytomy that does not conflict with the resolutions found in the other analyses. These two cases are as follows: (1) *Vitales* + *Saxifragales* supported by ≥ 70% BS in all analyses but RY, with *Saxifragales*, *Vitales*, and remaining *Rosidae* forming a polytomy in the RY tree (Figure 7); (2) *Dasypogonaceae* + *Arecales* in all but RY (52%, 78%, and 80% BS in the ntNo3rd, AA, and ntAll trees, respectively) and a trichotomy of *Dasypogonaceae*, *Arecales*, and *Poales* + (*Zingiberales* + *Commelinales*) in the RY tree (Figure 7). In two additional cases when RY is compared to the other three analyses, the RY analysis produced either stronger support for the placement of a taxon or a different placement altogether. First, in the ntAll, ntNo3rd, and AA analyses, the position of *Sabiaceace* among the early-diverging lineages of *Eudicotyledoneae* is weakly supported. However, in the RY analysis, *Sabiaceae* receive moderate support (79% BS; Figure 7) as sister to a strongly supported (100% BS; Figure 7) clade of *Trochodendrales* + (*Buxales* (*Gunnerales* + *Pentapetalae*)). This contrasts with previous studies that often place *Sabiaceae* as sister to *Proteales*[103]. An example of a different placement of a taxon in the RY analysis when compared to the other analyses involves *Liliales*. The ntAll, ntNo3rd, and the AA analyses support *Liliales* as sister to a clade of *Dioscoreales* + *Pandanales* with 72%, 69%, and 80% BS, respectively. This placement of *Liliales* was also recovered in Barrett et al. [108]. In contrast, in the RY analysis, *Liliales* are placed in a clade with *Asparagales* + *Commelinidae* with moderate support (69% BS; Figure 7). This latter placement of *Liliales* was strongly supported in an analysis with much better taxon sampling [103].

Some taxa that have been problematic in previous studies (e.g., *Boraginaceae, Ceratophyllales*, the COM clade, *Dilleniaceae*, and *Zygophyllaceae*) continue to defy definitive placement. Their positions vary among our analyses, although they are generally not well supported in some, or all, of the trees. Despite its general placement of the COM clade in *Fabidae* in these and other plastid analyses, this clade is more closely related to *Malvidae* in some analyses, particularly those using mitochondrial gene sequences (reviewed in [103]). Recent analyses of plastid, mitochondrial, and nuclear data suggest that the COM clade may represent ancient reticulation involving *Fabidae* and *Malvidae* during the rapid radiation of *Rosidae* (Sun et al., in prep.).

### Methodological issues of plastid phylogenomic analyses

To address potential systematic error in large-scale phylogenetic analyses, scientists often either try to improve the fit of models to the data or change or remove problematic data. With increasing sequence length and number of genes, it is more likely that a sequence alignment will contain regions with heterogeneous processes of molecular evolution. We see evidence of this high heterogeneity with our model-fitting experiments, which always favor the most parameter-rich models (Table 2). Thus, defining partitioning schemes and models that can accurately reflect the true processes of molecular evolution while not over-parameterizing the analysis remains critically important for phylogenetic analyses of large plastid data sets. Although we assessed models that account for heterogeneity in patterns of molecular evolution among genes and in some cases codon positions, our model selection tests only evaluated a small selection of possible models and partitioning schemes. It is possible that other partitioning schemes could enable simpler models.

Most conventional phylogenetic models, like those used in our analyses, also assume homogeneous processes of evolution throughout the tree. Yet when the branches of the phylogeny encompass over one billion years of evolutionary history, as likely do those in the green plants, the patterns of evolution almost certainly differ among lineages and through time. This is apparent from the often good fit of covarion models (which may better describe rate shifts through time) to plastid genes [109,110] and the presence of nucleotide compositional heterogeneity, which can confound conventional phylogenetic analyses (e.g., [111,112]). Also, our models do not account for shifts in selective pressure or instances of positive selection that will affect nucleotide and amino acid substitution patterns (e.g., [113,114]).

Nucleotide compositional heterogeneity remains a concern for green plant plastid genome analyses. This variation is most evident in non-seed plant taxa (Figure 2), and thus it has not been a focus of many previous phylogenetic analyses of plastid genome sequences. A GC bias in itself is not necessarily problematic for phylogenetic analyses, but nearly all commonly used models for likelihood-based phylogenetic analyses assume single equilibrium nucleotide frequencies. Given that GC content appears to vary by codon position in plants (Figures 1 and 2) [115-117], a partitioning scheme that estimates separate nucleotide frequencies for each codon position may account for some of the spatial heterogeneity in GC content in the plastid genome, but it does not address the differences in GC frequency among lineages.

A commonly used strategy to reduce the effects of GC heterogeneity across lineages is RY-coding, in which the purines (A and G) are coded as Rs and the pyrimidines (C and T) are coded as Ys [118]. RY-coding can reduce the compositional variability among lineages, improve the fit of models, and increase the signal for internal branches [118-121]. An obvious disadvantage to RY-coding is that by coding the sequences with two character states instead of four, it reduces the amount of information in the sequences. In general, we see little overall reduction, and even some gains, in bootstrap support when using RY-coding compared to the use of all nucleotide data (ntAll), suggesting that the benefits of RY-coding make up for any potential costs of information loss. Perhaps the biggest topological difference in the RY phylogeny (Figure 7) compared to ntAll (Figure 5) is the placement of *Monilophyta* rather than *Lycopodiophyta* as sister to seed plants. The unexpected placement of *Lycopodiophyta* as the sister to seed plants in the ntAll analysis (Figure 5) is almost certainly an artifact of systematic error; several other lines of evidence support *Monilophyta* as the sister group of seed plants (see above).

Approaches to reducing systematic errors by excluding problematic data, which often include fast-evolving or saturated sites, also have been suggested for plastid genome analyses [20,41,80,110,122]. With the proper model of molecular evolution and adequate taxon sampling, fast sites are not necessarily problematic; they are only problematic insofar as they are difficult to model. Yet with heterogeneous processes of molecular evolution throughout the tree, the fast-evolving or saturated sites can produce a significant non-phylogenetic signal (e.g., [123]). Indeed, the third codon positions appear to have especially high levels of compositional heterogeneity, potentially causing systematic error (Figures 1 and 2), and an analysis of just the third codon positions (nt3rdOnly) conflicts with the analyses of other data sets in several critical parts of the tree (Additional file 11). However, third codon positions also represent a large proportion of the variable sites in the alignment, and removing them may exclude much of the phylogenetic information in some parts of the tree. With regard to backbone relationships in our phylogeny, excluding the third position sites (ntNo3rd) produces several interesting changes in contrast to ntAll: 1) it supports the sister relationship of mosses and liverworts, 2) monilophytes, not lycophytes, are placed sister to seed plants as expected, and 3) support for some of the backbone angiosperm relationships is reduced. Thus, the effects of removing the third codon position sites appear to vary in different parts of the tree.

Another strategy for overcoming potential error associated with fast-evolving sites is to code the sequences as amino acids rather than nucleotides. This does not necessarily eliminate problems of compositional heterogeneity, as the GC bias also may bias amino acid composition (Figures 3 and 4) [124]. Regarding backbone green plant relationships, the AA analysis provided similar results to analyses of only first and second codon positions. AA analysis also produced some weakly supported, questionable relationships among angiosperm lineages (i.e., *Piperales* + *Ceratophyllales*; Figure 8). In previous deep-level plant analyses, analyses of amino acid data have resulted in arguably more problematic or questionable relationships than analyses of nucleotide data [29,80]. However, these results are likely due to inappropriate models of amino acid evolution [125], and with better models, optimized for plastid evolution, amino acid data may be a valuable source of phylogenetic information.

Taxon sampling is also important for plastid phylogenomic studies, especially when the model of evolution is inadequate [56,58,126-131], and genome-scale analyses often have limited taxon sampling. New methods for rapid and inexpensive plastid genome sequencing (e.g., [132]) may ameliorate the effects of insufficient sampling of extant taxa; however, many major lineages of green plants are now extinct, precluding their inclusion in analyses of molecular data (but see [133-136]). In addition, ancient, rapid radiations abound within portions of the green plant tree of life, creating extremely difficult phylogenetic problems no matter the taxon sampling [63,69,107,137].

Furthermore, even in the absence of systematic error, it is possible that a tree built from plastid genome data will not reflect species relationships. The plastid genome represents a single locus of linked genes (i.e., a single coalescent history). For phylogenetic analyses, this can be beneficial because combining genes with different evolutionary histories into a single character matrix can lead to phylogenetic error [138-140]. However, incomplete lineage sorting or ancient reticulation could lead to conflict between the plastid gene tree and the species phylogeny [141]. For this reason, it will be interesting to compare phylogenetic hypotheses from the plastid genome with independent phylogenetic estimates from numerous nuclear and mitochondrial loci.

Finally, while full plastid genome sequence data provide much power for resolving difficult phylogenetic relationships, it is not clear that they can resolve all plant relationships. Theoretical work suggests that extremely large data sets may be necessary to resolve some relationships when the internal nodes are separated by very short branches [142], and recent analyses indicate that full plastid genomes are not sufficient to reject alternative topologies among monocots [108]. Indeed, the unresolved or conflicting parts of the green plant phylogeny in our analyses are generally associated with short internal branch lengths (see Additional files 7, 8, 9, 10, and 11). Thus, even if the model of evolution accurately reflects the true process of molecular evolution, and there is no systematic error, plastid genome data alone may not be sufficient to resolve all parts of the green plant tree of life. That is, the topology may not be identifiable with the plastid data alone. A recent analysis using a new diagnostic test for phylogenetic identifiability based on data cloning suggested that a backbone topology of angiosperms was identifiable from plastid sequence data using the GTR + Γ model [143], but the tree in this paper is much larger and the models more complex. In any case, it will be necessary to include perspectives from the nuclear genome and phenotypic data before we are confident about all deep-level relationships among green plants.

## Conclusions

Our diverse analyses provide a first approach to addressing some of the difficult issues associated with plastid phylogenetic analyses at this evolutionary depth and level of taxon sampling. The results of the analyses using different models, character-coding strategies, and character subsets suggest that much of the tree is robust to many different phylogenetic approaches, and they highlight regions of the tree that need more scrutiny (i.e., those relationships not consistent across analyses). More sophisticated modelling approaches may more accurately characterize the heterogeneous processes of molecular evolution, but it is also crucial that the parameters of these complex models can be estimated by the data at hand [143]. While it may be impossible for any model to reflect perfectly the complexities of molecular evolution, as we better characterize these processes it will be possible to examine through simulations their possible effects on phylogenetic analyses and to recognize phylogenetic error caused by model misspecification.

## Methods

### Taxon and sequence sampling

Protein-coding data, including nucleotides and their corresponding amino acid sequences, for all *Viridiplantae* taxa that had complete or nearly complete plastid genome sequences were downloaded from GenBank on February 28, 2012. If there were multiple genome sequences from the same taxon, we included the sequence with the most data. Our sampling included most major lineages of *Viridiplantae*. A complete list of taxa and GenBank accession numbers is available in Additional file 1.

Taxonomic names (Additional file 1) follow various references. Four classes of chlorophytic algae (*Chlorophyta*) are recognized following a traditional classification [26,76]. Classes of streptophytic algae and orders for both chlorophytic and streptophytic algae follow Leliaert et al. [76]. Names for the three main bryophyte clades follow recent classifications: mosses (*Bryophyta*[144]), hornworts (*Anthocerotophyta*[145]), and liverworts (*Marchantiophyta*[146]). Major clades of tracheophytes follow Cantino et al. [147] and Soltis et al. [103]. Familial and ordinal names within major clades of land plants follow these references: *Bryophyta*[144]; *Anthocerotophyta*[145]; *Marchantiophyta*[146]; lycophytes (*Lycopodiophyta*) and ferns (*Monilophyta*) [148]; gymnosperms (*Acrogymnospermae*[149]); and angiosperms (*Angiospermae*[150]). All scientific names are italicized to distinguish common names from scientific names [147,151].

### Building the phylogenetic character matrix

To build the phylogenetic matrix, first we used a clustering approach to identify homologous gene sequences. Amino acid sequences from all downloaded genomes were compared to each other using BLASTP v.2.2.26 [152]. Significant BLAST hits were defined as those having a maximum *e*-value of 1.0e^-5^ and having the hit region cover at least 40% of the target and query sequences. Based on the BLAST hits, we formed clusters of putative homologs using single-linkage clustering. This approach identified groups of sequences that had a significant BLAST hit with at least one other sequence in the cluster and were connected to each other by a path of significant BLAST hits. The resulting clusters were modified in two ways. First, clusters that contained two or more different genes from a single taxon were re-clustered at a more stringent *e*-value to separate the genes. Second, when it appeared that a single gene was split into multiple clusters, we combined them. Some clusters contained multiple sequences from the same species when the gene was present in the inverted repeat region in the plastid genome. If the sequences were identical, only one was retained for analysis. In cases where the two sequences differed slightly, we removed both sequences. Only clusters containing sequences from at least 50% of the 360 taxa were retained for the phylogenetic analyses.

Each remaining amino acid cluster (78 total) was aligned with MAFFT v. 6.859 [153] using the L-INS-i algorithm, and subsequently, poorly aligned regions were removed using trimAl v.1.2rev59 [154]. After using trimAl, we also visually inspected the trimmed alignments and removed poorly aligned regions. The nucleotide sequences for each cluster were aligned with PAL2NAL v.14 [155] to correspond to the trimmed amino acid alignment and ensure that the correct reading frame was maintained. We checked for anomalous sequences by building ML trees from each of the aligned clusters with RAxML [156,157] following the search strategies outlined below. These topologies were visually examined, and sequences in obviously spurious locations in the tree were removed. If any sequences were removed from a cluster alignment, we realigned and edited the cluster’s untrimmed data as described above. Alignments for each gene were concatenated using FASconCAT v.1.0 [158].

From this data set, we generated an amino acid (AA) alignment, two nucleotide alignments, and a binary character alignment. The first nucleotide alignment contained all nucleotide positions (ntAll), while the second contained only the first and second codon positions (ntNo3rd). The binary character alignment was an RY-coded version (RY) of the ntAll data set. RY-coding [159] involves recoding the nucleotides as binary characters, either purines (A or G = R) or pyrimidines (C or T = Y). RY-coding has been used to ameliorate biases caused by saturation, rate heterogeneity, and base composition [119,160,161]. To determine if the data sets were decisive using our selected partitioning schemes (see below), we followed the approach used in Sanderson et al. [72].

We assessed base composition bias in the nucleotide data set (ntAll) by conducting a chi-square test using PAUP* v.4.0b10 [162] to determine if the base frequencies across taxa were homogeneous. To determine if base composition of the nucleotide sequences in the ntAll matrix could affect the composition of amino acid sequences in the AA matrix, we conducted linear regressions in R [163]. We examined the relationship of percent GC content to the percent of amino acids that are coded for by GC-rich codons (i.e., G, A, R, and P) as well as the relationship of percent GC content to the percent of amino acids that are coded for by AT-rich codons (i.e., F, Y, M, I, N, and K).

### Phylogenetic analyses

All ML phylogenetic analyses were implemented with RAxML v. 7.3.0 [156,157]. The optimal partitioning scheme for each alignment was chosen from among several commonly used partitioning strategies using the corrected Akaike information criterion (AICc) [164,165]. This penalizes models for additional parameters and should account for the trade-off between increased model fit and over-parameterization when choosing the best model. For the nucleotide (ntAll and ntNo3rd) and RY-coded data, we examined four possible partitioning strategies: 1) no partitioning, 2) partitioning by each codon position (three partitions), 3) partitioning by gene (78 partitions), and 4) partitioning by each codon position within each gene (234 partitions). For the AA data, we tested two partitioning strategies: 1) no partitioning, and 2) partitioning by gene (78 partitions). A novel approach for determining partitions of phylogenomic data sets a posteriori using a Bayesian mixture model has recently been proposed [69]. Additionally, the program PartitionFinder [166] allows for the statistical comparison of multiple a priori partitioning schemes. We explored both of these methods, but we were unable to complete the analyses due to computational limitations resulting from the large size of our data set.

To determine which partitioning scheme was optimal for each data set, we first obtained the optimal ML tree for each data set under each partitioning scheme as follows. For the nucleotide (ntAll, ntNo3rd) and RY-coded data, we ran 10 ML searches from different starting trees. We used the GTR+Γ model of evolution for each partition in the nucleotide data set and the binary model of evolution (BINGAMMA) for the RY data set. For the AA data, we ran 3 ML searches from different starting trees. To select the best amino acid substitution model for each partition of the AA data set, we used the Perl script (ProteinModelSelection.pl) included in the RAxML distribution package. For each ML search, we estimated a separate substitution rate matrix for each partition but a single set of branch length parameters for all partitions. We then optimized the model and branch lengths on each resulting ML tree using RAxML (-f e). AICc values for each partitioning scheme were then calculated by using the log-likelihood, number of estimable parameters, and sample size given by RAxML. The optimal partitioning strategy for each data set was then used in subsequent ML bootstrap analyses. Bootstrap searches (200 replicates for each matrix) were executed separately from the search for the best ML tree using the standard bootstrap option in RAxML. To determine if 200 replicates were adequate for estimating bootstrap values, we conducted a posteriori bootstopping analyses (-I autoMRE) as implemented in RAxML and described in Pattengale et al. [167]. All trees were rooted at the branch between *Chlorophyta* and *Streptophyta*[23,24].

To further explore our data, we conducted the following phylogenetic analyses using the methods described above unless otherwise noted. To determine if there is conflict between the phylogenetic signal in the ntNo3rd data set and the data set containing only third positions (nt3rdOnly), we analyzed the nt3rdOnly data partitioned by gene region. We also conducted phylogenetic analyses on each of the four main data sets (ntAll, ntNo3rd, RY, and AA) with four taxa removed: *Neottia nidus-avis* and *Rhizanthella gardneri* (mycoheterotrophic orchids), *Epifagus virginiana* (a parasitic flowering plant), and *Helicosporidium* sp. (a parasitic green alga). These taxa have elevated rates of molecular evolution and relatively few genes present in the data sets (see Additional file 2). We removed them to ensure that their inclusion did not cause any phylogenetic artifacts.

## Availability of supporting data

The data sets supporting the results of this article are available in the Dryad Digital Repository: http://doi.org/10.5061/dryad.k1t1f.

## Competing interests

The authors declare that they have no competing interests.

## Authors’ contributions

BRR conceived the study. BRR, PSS, DES, and JGB participated in the design of the study. BRR, MAG, and JGB analyzed the data. BRR, PSS, DES, and JGB wrote the paper. All authors read and approved the final manuscript.

## Supplementary Material

Additional file 1 **Taxon sampling.** Taxa included in this study, their GenBank accession numbers, original publications, and their higher taxonomy. Click here for file

Additional file 2 **Genes sampled and missing data for each taxon.** Information on taxa sampled for each gene included, and the percent of missing data for each taxon in each data set. Number of genes present per taxon and number of taxa present per gene are also given. Click here for file

Additional file 3 GC content for each taxon in the ntAll and ntNo3rd data sets as well as in the first, second, and third codon positions of the ntAll data set. Click here for file

Additional file 4 **Fifty percent maximum likelihood majority-rule bootstrap consensus summary tree of *Viridiplantae *** **inferred from the first and second codon positions (ntNo3rd) analysis.** See also Figure 6 for a summary tree of major *Viridiplantae* clades and Additional file 1 for taxonomy. Data set derived from 78 protein-coding genes of the plastid genome (ntax = 360, 38,898 bp, missing data ~15.6%,). Bootstrap support values ≥ 50% are indicated. Click here for file

Additional file 5 **Fifty percent maximum likelihood majority-rule bootstrap consensus tree of *Viridiplantae *** **inferred from the RY-coded (RY) analysis.** See also Figure 7 for a summary tree of major *Viridiplantae* clades and Additional file 1 for taxonomy. Data set derived from 78 protein-coding genes of the plastid genome (ntax = 360, 58,347 bp, missing data ~15.6%,). Bootstrap support values ≥ 50% are indicated. Click here for file

Additional file 6 **Fifty percent maximum likelihood majority-rule bootstrap consensus tree of *Viridiplantae *** **inferred from the amino acid (AA) analysis.** See also Figure 8 for a summary tree of major *Viridiplantae* clades and Additional file 1 for taxonomy. Data set derived from 78 protein-coding genes of the plastid genome (ntax = 360, 19,449 AAs, missing data ~15.6%,). Bootstrap support values ≥ 50% are indicated. Click here for file

Additional file 7 **Maximum likelihood tree of *Viridiplantae *** **inferred from the all nucleotide positions (ntAll) analysis.** Cladogram of the maximum likelihood bipartition tree is shown on the left with bootstrap values indicated above the branches. The phylogram of same tree is shown on the right. Data set derived from 78 protein-coding genes of the plastid genome (ntax = 360; 58,347 bp; missing data ~15.6%). Bootstrap support values ≥ 50% are indicated. Click here for file

Additional file 8 **Maximum likelihood tree of *Viridiplantae *** **inferred from the first and second codon positions (ntNo3rd) analysis.** Cladogram of the maximum likelihood bipartition tree is shown on the left with bootstrap values indicated above the branches. The phylogram of same tree is shown on the right. Data set derived from 78 protein-coding genes of the plastid genome (ntax = 360, 38,898 bp, missing data ~15.6%,). Bootstrap support values ≥ 50% are indicated. Click here for file

Additional file 9 **Maximum likelihood tree of *Viridiplantae *** **inferred from the RY-coded (RY) analysis.** Cladogram of the maximum likelihood bipartition tree is shown on the left with bootstrap values indicated above the branches. The phylogram of same tree is shown on the right. Data set derived from 78 protein-coding genes of the plastid genome (ntax = 360, 58,347 bp, missing data ~15.6%,). Bootstrap support values ≥ 50% are indicated. Click here for file

Additional file 10 **Maximum likelihood tree of *Viridiplantae *** **inferred from the amino acid (AA) analysis.** Cladogram of the maximum likelihood bipartition tree is shown on the left with bootstrap values indicated above the branches. The phylogram of same tree is shown on the right. Data set derived from 78 protein-coding genes of the plastid genome (ntax = 360, 19,449 AAs, missing data ~15.6%,). Bootstrap support values ≥ 50% are indicated. Click here for file

Additional file 11 **Maximum likelihood tree of *Viridiplantae *** **inferred from the third codon position (nt3rdOnly) analysis.** Cladogram of the maximum likelihood bipartition tree is shown on the left with bootstrap values indicated above the branches. The phylogram of same tree is shown on the right. Data set derived from 78 protein-coding genes of the plastid genome (ntax = 360, 19,449 bp, missing data ~15.6%,). Bootstrap support values ≥ 50% are indicated. Click here for file
