# Supplementary figures and images for: From algae to angiosperms–inferring the phylogeny of green plants (Viridiplantae) from 360 plastid genomes
Source: BMC Evol Biol. 2014 Feb 17;14:23. doi: 10.1186/1471-2148-14-23 (PMC3933183; doi:10.1186/1471-2148-14-23)

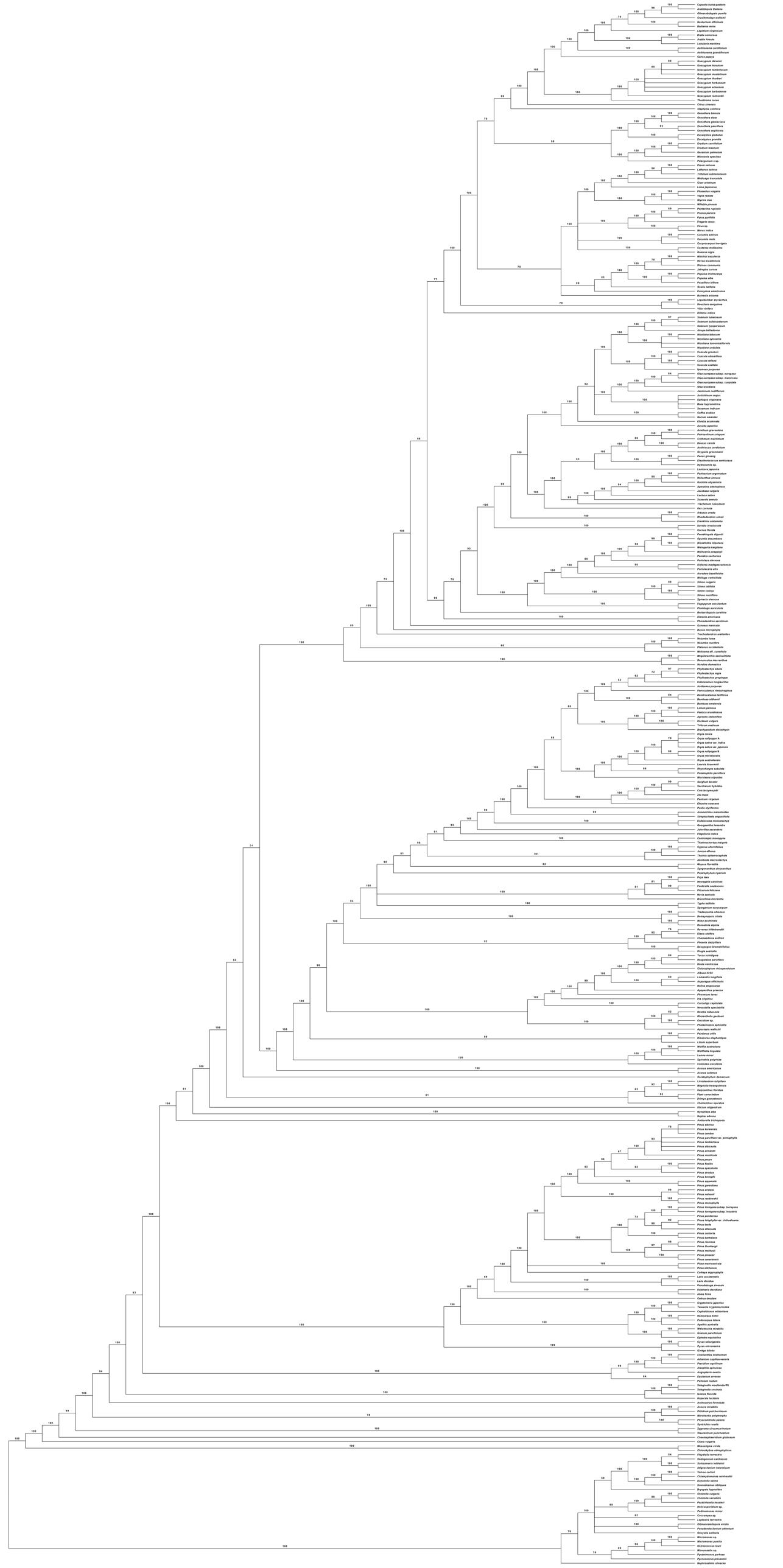

Supplement: Additional file 4 — Fifty percent maximum likelihood majority-rule bootstrap consensus summary tree of Viridiplantae inferred from the first and second codon positions (ntNo3rd) analysis. See also Figure 6 for a summary tree of major Viridiplantae clades and Additional file 1 for taxonomy. Data set derived from 78 protein-coding genes of the plastid genome (ntax = 360, 38,898 bp, missing data ~15.6%,). Bootstrap support values ≥ 50% are indicated. [file 1471-2148-14-23-S4.pdf]

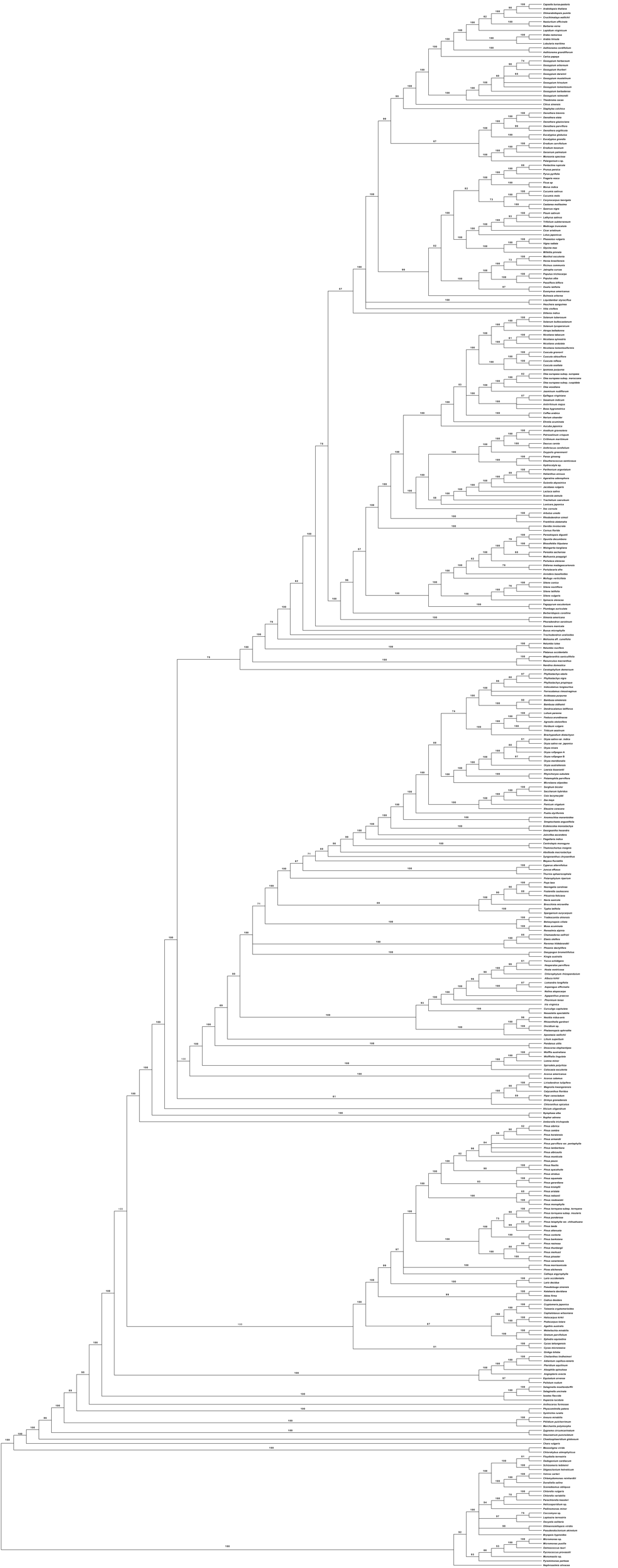

Supplement: Additional file 5 — Fifty percent maximum likelihood majority-rule bootstrap consensus tree of Viridiplantae inferred from the RY-coded (RY) analysis. See also Figure 7 for a summary tree of major Viridiplantae clades and Additional file 1 for taxonomy. Data set derived from 78 protein-coding genes of the plastid genome (ntax = 360, 58,347 bp, missing data ~15.6%,). Bootstrap support values ≥ 50% are indicated. [file 1471-2148-14-23-S5.pdf]

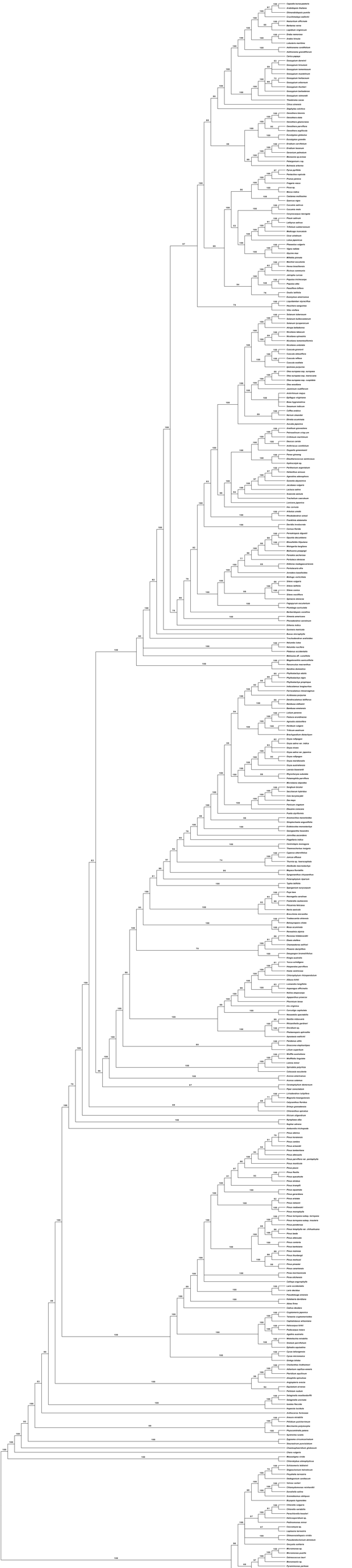

Supplement: Additional file 6 — Fifty percent maximum likelihood majority-rule bootstrap consensus tree of Viridiplantae inferred from the amino acid (AA) analysis. See also Figure 8 for a summary tree of major Viridiplantae clades and Additional file 1 for taxonomy. Data set derived from 78 protein-coding genes of the plastid genome (ntax = 360, 19,449 AAs, missing data ~15.6%,). Bootstrap support values ≥ 50% are indicated. [file 1471-2148-14-23-S6.pdf]

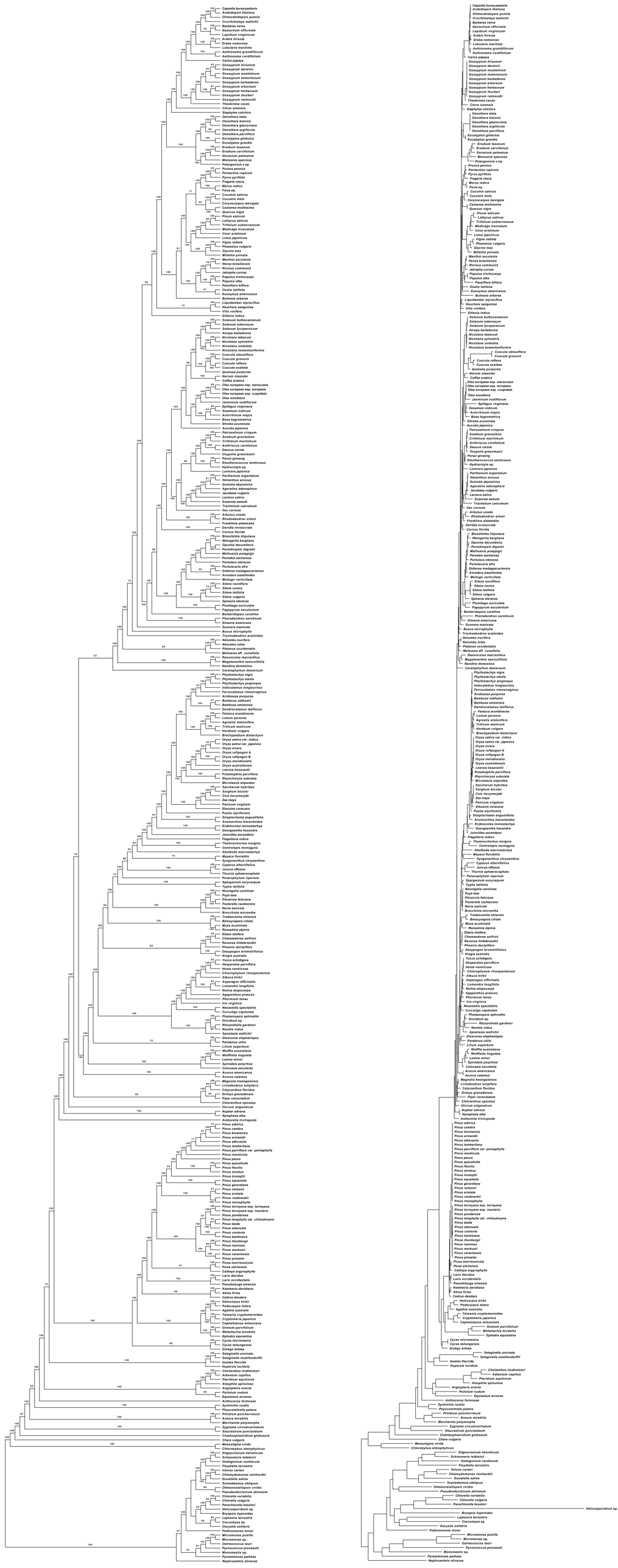

Supplement: Additional file 7 — Maximum likelihood tree of Viridiplantae inferred from the all nucleotide positions (ntAll) analysis. Cladogram of the maximum likelihood bipartition tree is shown on the left with bootstrap values indicated above the branches. The phylogram of same tree is shown on the right. Data set derived from 78 protein-coding genes of the plastid genome (ntax = 360; 58,347 bp; missing data ~15.6%). Bootstrap support values ≥ 50% are indicated. [file 1471-2148-14-23-S7.pdf]

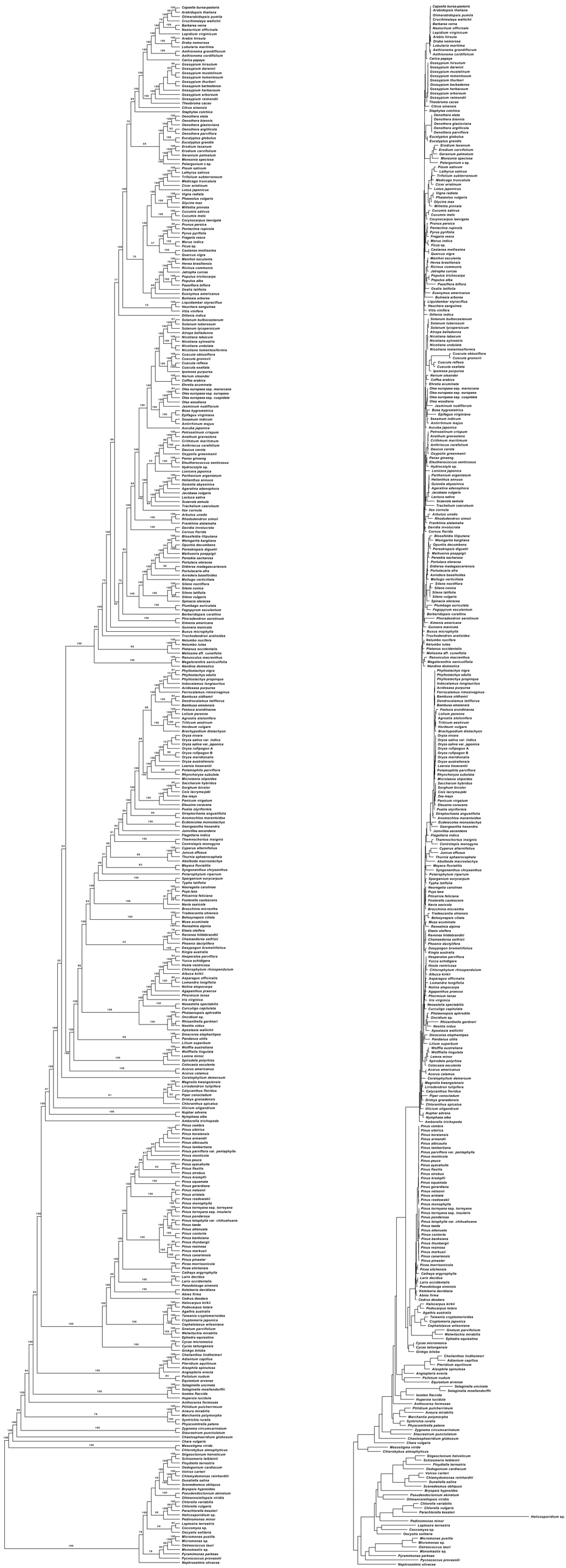

Supplement: Additional file 8 — Maximum likelihood tree of Viridiplantae inferred from the first and second codon positions (ntNo3rd) analysis. Cladogram of the maximum likelihood bipartition tree is shown on the left with bootstrap values indicated above the branches. The phylogram of same tree is shown on the right. Data set derived from 78 protein-coding genes of the plastid genome (ntax = 360, 38,898 bp, missing data ~15.6%,). Bootstrap support values ≥ 50% are indicated. [file 1471-2148-14-23-S8.pdf]

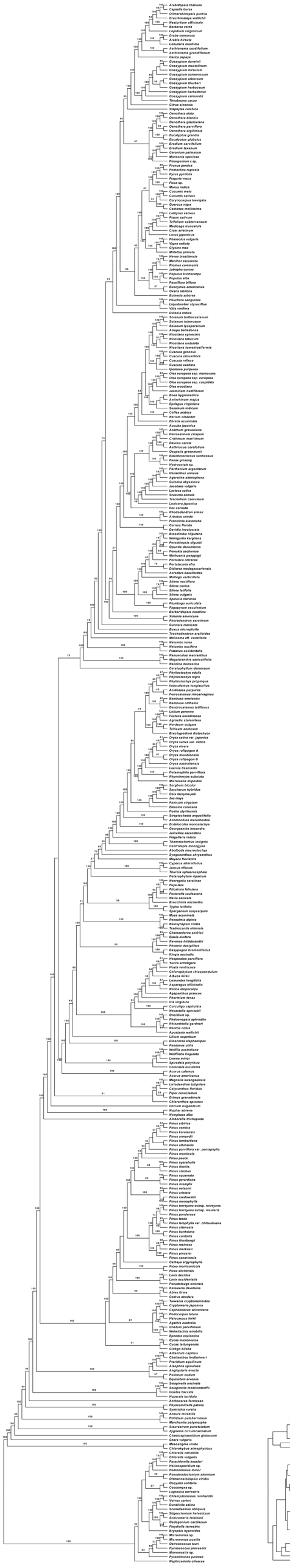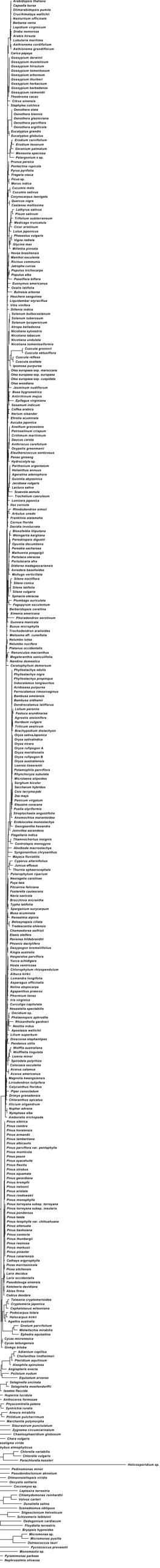

Supplement: Additional file 9 — Maximum likelihood tree of Viridiplantae inferred from the RY-coded (RY) analysis. Cladogram of the maximum likelihood bipartition tree is shown on the left with bootstrap values indicated above the branches. The phylogram of same tree is shown on the right. Data set derived from 78 protein-coding genes of the plastid genome (ntax = 360, 58,347 bp, missing data ~15.6%,). Bootstrap support values ≥ 50% are indicated. [file 1471-2148-14-23-S9.pdf]

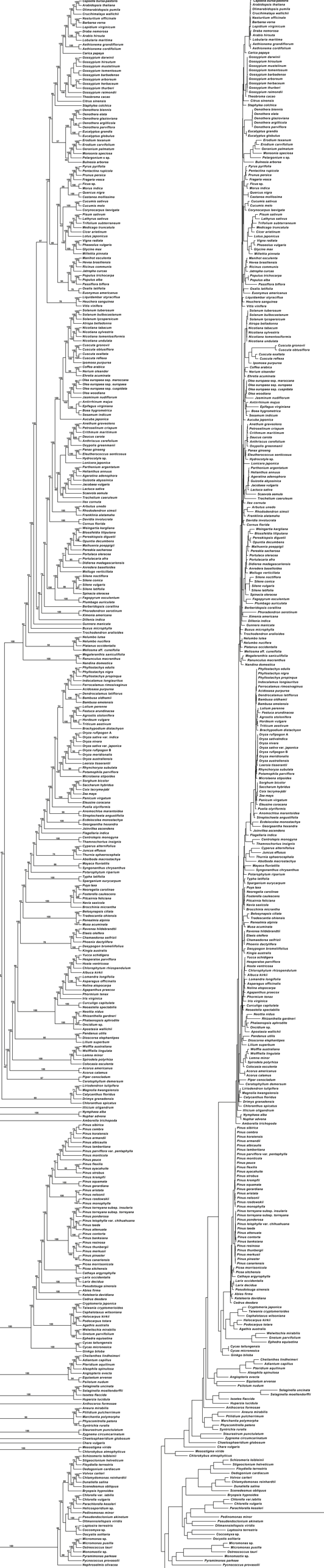

Supplement: Additional file 10 — Maximum likelihood tree of Viridiplantae inferred from the amino acid (AA) analysis. Cladogram of the maximum likelihood bipartition tree is shown on the left with bootstrap values indicated above the branches. The phylogram of same tree is shown on the right. Data set derived from 78 protein-coding genes of the plastid genome (ntax = 360, 19,449 AAs, missing data ~15.6%,). Bootstrap support values ≥ 50% are indicated. [file 1471-2148-14-23-S10.pdf]

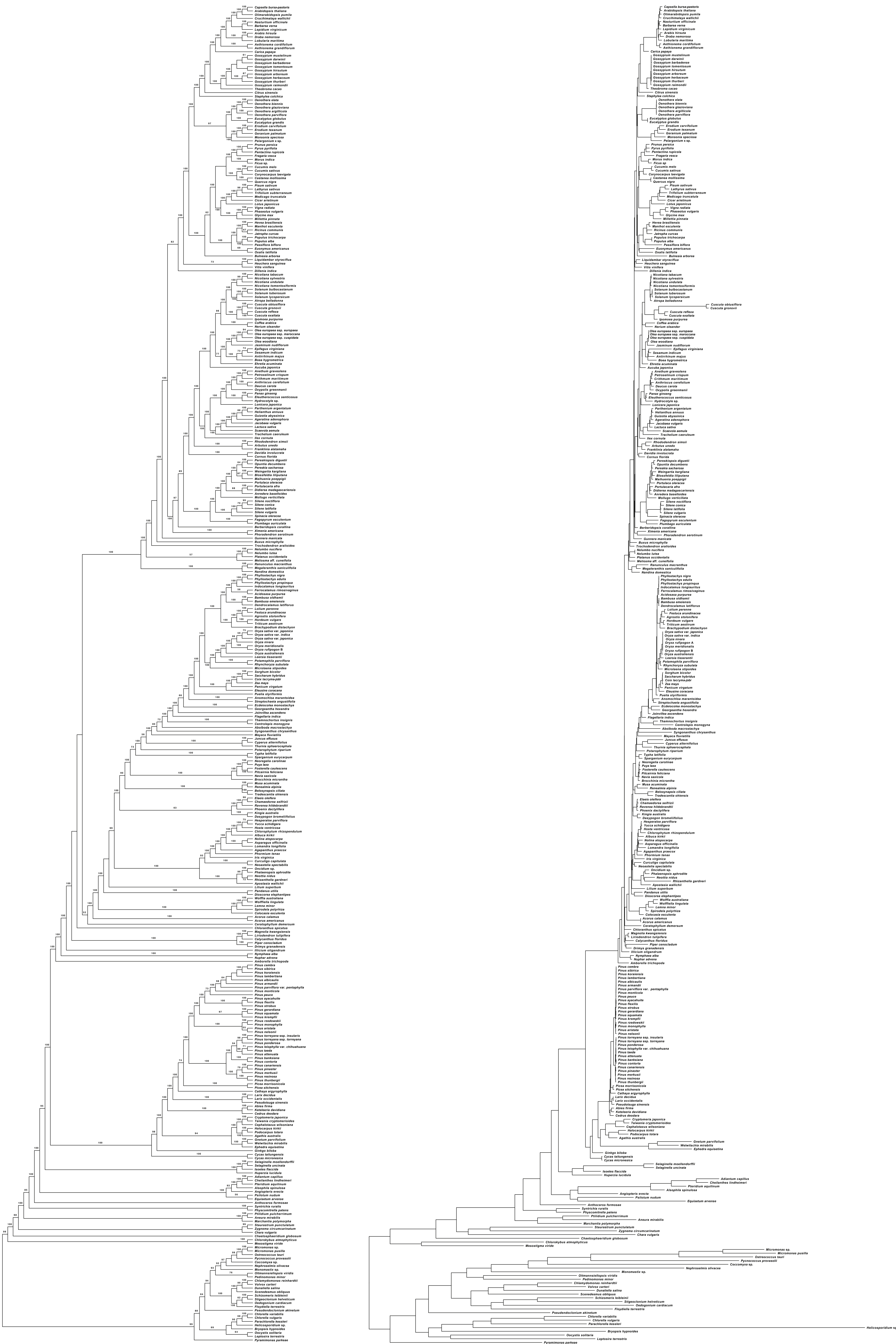

Supplement: Additional file 11 — Maximum likelihood tree of Viridiplantae inferred from the third codon position (nt3rdOnly) analysis. Cladogram of the maximum likelihood bipartition tree is shown on the left with bootstrap values indicated above the branches. The phylogram of same tree is shown on the right. Data set derived from 78 protein-coding genes of the plastid genome (ntax = 360, 19,449 bp, missing data ~15.6%,). Bootstrap support values ≥ 50% are indicated. [file 1471-2148-14-23-S11.pdf]
